# Prevalence and Genotype Distribution of Sapovirus in China: A Systematic Review and Meta‐Analysis

**DOI:** 10.1002/gch2.202400369

**Published:** 2025-08-05

**Authors:** Zhao Gao, Xiu‐jie Qin, Ting‐jun Li, Xue‐qiang Sun, Hui Zhang, Shan‐shan Pan, Ting‐ting Qiu

**Affiliations:** ^1^ Department of Clinical Laboratory Jinan Second People's Hospital Jinan Shandong China; ^2^ Department of Surgery Emergency Jiaozhou Hospital East Hospital Affiliated to Tongji University Qingdao Shandong China; ^3^ Department of Prevention of Infectious Diseases Xi'an Center for Disease Control and Prevention Xi'an Shaanxi China; ^4^ Department of Catheter Room Jiaozhou Hospital East Hospital Affiliated to Tongji University Qingdao Shandong China

**Keywords:** acute gastroenteritis, disease burden, meta‐analysis, prevalence, sapovirus

## Abstract

Sapovirus is gaining recognition as a significant non‐bacterial causative agent of acute gastroenteritis globally, contributing to both sporadic cases and outbreaks across all age groups. In China, it is identified as the second most prevalent pathogen responsible for acute gastroenteritis outbreaks, following norovirus, which underscores its public health importance. Consequently, an extensive systematic review and meta‐analysis are undertaken to evaluate the prevalence and genotype distribution of sapovirus among patients presenting with acute gastroenteritis. This analysis incorporated 159 eligible studies spanning 32 provinces in China. The estimated overall prevalence of sapovirus is 1.9% (95% CI: 1.7–2.2), with an asymptomatic prevalence of 0.8% (95% CI: 0–2.5). Notably, in outbreak settings, the respective prevalence rates increase substantially to 16.4% (95% CI: 10.1–23.8) and 14.4% (95% CI: 8.9–20.7). Furthermore, these findings reveal that sapovirus GI genomes predominated in both sporadic and outbreak contexts, with genotypes GI.1, GI.2, and GII.1 being most frequently identified. These insights are crucial for enabling governments to accurately assess disease burden, inform the development of targeted vaccines, and establish evidence‐based public health policies and emergency response strategies to mitigate sapovirus outbreaks.

## Graphical abstract

This review highlights a distinct age‐related trend in Sapovirus (SaV) prevalence in China, peaking among kindergarten children and adults. These results not only enhance the understanding of SaV's epidemiology but also provide a robust foundation for estimating disease burden and informing the development of targeted vaccines and public health policies.

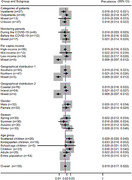

## 1 Introduction

Acute gastroenteritis (AGE) is a highly infectious disease marked by acute diarrhea and vomiting, posing a significant threat to global health and accounting for considerable mortality, particularly responsible for 9% of fatalities among children under five years of age.^[^ ^1^, ^2^ ^]^ Sapovirus (SaV), a prominent pathogen of non‐bacterial AGE, is known to cause both sporadic cases and outbreaks across all age groups worldwide, with a notable impact on infants.^[^ ^3^ ^]^ While most studies indicate that SaV‐induced AGE tends to be less severe than norovirus (NoV)‐associated AGE—both pathogens belonging to the human Caliciviridae family—recent literature has documented instances of SaV infection leading to hospitalization and severe dehydration.^[^ ^3^, ^4^, ^5^ ^]^ Moreover, research has uncovered an association between SaV infection and poor cognitive performance in children within low‐ and middle‐income countries.^[^ ^6^ ^]^ Over the past decade, there has been a marked rise in both sporadic cases and outbreaks attributable to SaV globally. However, the prevalence of SaV exhibits significant geographical variation, ranging from 1% to 17% across different regions.^[^ ^7^, ^8^ ^]^ In nations where rotavirus vaccines have been incorporated into national immunization programs, a notable increase in SaV prevalence among children with AGE has been observed, with rates escalating from 1.4% to 7.1% and from 1.9% to 3.4%.^[^ ^9^, ^10^ ^]^ Cross‐country studies have even demonstrated that in eight distinct low‐income countries, SaV exhibits a higher prevalence than rotavirus, adenovirus, NoV, or astrovirus among children with AGE.^[^ ^11^ ^]^ Consequently, SaV infection has emerged as a significant global public health concern and is increasingly recognized for its substantial contribution to the burden of AGE.^[^ ^12^ ^]^

Prior to the widespread adoption of real‐time reverse‐transcription polymerase chain reaction assays for viral detection in clinical specimens around 1997, distinguishing between NoV and SaV through laboratory diagnosis was nearly impossible.^[^ ^13^ ^]^ Over the past decade, as many countries have increasingly employed more sensitive molecular techniques, SaV has emerged as a key etiological agent in both sporadic cases and outbreaks of AGE.^[^ ^1^, ^13^, ^14^ ^]^ This extensive use of advanced methods has equipped researchers with a wider range of data to assess the disease burden of SaV‐related acute gastroenteritis. Several systematic assessments have been carried out to determine the prevalence of SaV in sporadic AGE cases.^[^ ^1^, ^15^, ^16^ ^]^ However, there is a lack of documented reports on the prevalence of SaV in outbreaks and asymptomatic infections among healthy individuals or those involved in outbreaks. In China, none of the aforementioned structured public data has been reported, and the population prevalence of SaV remains unclear. To our knowledge, this study represents the first comprehensive systematic review and meta‐analysis of the prevalence and genotype distribution of SaV in China. Our primary objective was to summarize the overall prevalence of SaV in sporadic and outbreak‐associated AGE patients and evaluate it using various subgroup variables (geographic region, patient type, monitoring periods, monitoring seasons, patient gender, patient age, and per capita income) among sporadic AGE patients. As a secondary objective, we aimed to assess the asymptomatic prevalence of SaV in healthy controls and healthy individuals involved in outbreaks. Based on these analyses, we also conducted an initial exploration of the molecular epidemiological characteristics of SaV.

## 2 Experimental Section

This systematic review and meta‐analysis complied with the updated preferred reporting items for systematic reviews and meta‐analyses (PRISMA) 2020 reporting guidelines.^[^ ^17^ ^]^ The article selection process was conducted in accordance with the revised PRISMA standard protocol. This study protocol was not registered in the PROSPERO international database.

### 2.1 Search Strategy

The literature search was executed across multiple databases, including PubMed, Web of Science, Medline, Cochrane Library, and four major Chinese full‐text databases: PubScholar (https://pubscholar.cn/), Wanfang (http://med.wanfangdata.com.cn/), China National Knowledge Infrastructure (CNKI, https://www.cnki.net/), and Weipu (http://www.cqvip.com/), to retrieve all articles on the prevalence of SaV from the inception of these databases to August 1, 2024. The search utilized keywords such as “Sapovirus”, “Sapovirus*”, “Taiwan”, “Hong Kong”, “Macao”, and “China”, tailored to the specific requirements of each search engine. In addition to the database search, the authors performed a comprehensive review of the references in the full‐text articles, as well as those cited in reviews, opinion pieces, and editorial articles, to identify additional relevant studies. The systematic review imposed no language restrictions; for articles not in English or Chinese, translation software was employed to extract the necessary information. Additional File S1 (Supporting Information) illustrates the detailed retrieval procedure for this study, using the PubMed and Web of Science databases as examples.

### 2.2 Selection Criteria

Two reviewers independently performed an initial screening of the eligible articles. Initially, articles from Chinese journals not indexed in China Core Journals or China Key Journals of Science and Technology, excluding dissertations, were excluded owing to potential concerns about research quality. Subsequently, the two reviewers carefully assessed the remaining articles based on their titles and abstracts. Articles were excluded at this stage if they met any of the following criteria: 1) the research was not conducted in China; 2) the subject was the molecular characteristics of SaV; 3) the focus was on SaV testing methods; 4) the study involved testing for pathogens other than SaV; 5) the subjects were animals, sewage, or non‐human entities; 6) the subjects were immunocompromised populations, such as HIV‐positive individuals or organ transplant recipients; or 7) the article was a review, opinion piece, letter to the editor, or editorial.

The two reviewers then carefully read the full text of the remaining articles and ultimately identified those that met the inclusion criteria. During this stage, the excluded articles exhibited the following characteristics: 1) SaV surveillance duration was less than one year; 2) SaV prevalence data were either uncalculable or unreported; 3) SaV testing was conducted exclusively on samples previously screened for other pathogens; 4) Non‐PCR‐based methods were used for SaV detection; 5) Duplicate data were identified across multiple articles, with only the most comprehensive one retained; 6) Full‐text access to certain articles was unavailable; 7) Articles were deemed to be of low quality. In the event of discrepancies between the two reviewers regarding article inclusion, a third independent reviewer was consulted to make the final determination.

### 2.3 Data Extraction

The criteria for AGE in this study were established as vomiting ≥2 times per day and/or defecation ≥3 times per day, accompanied by abnormal stool characteristics, such as loose, watery, mucous, bloody, or bloody stools.^[^ ^18^ ^]^ The prevalence of SaV was calculated as the number of AGE cases that tested positive for SaV in stool or other samples divided by the total number of AGE cases assessed via PCR‐based methods. To capture all SaV cases, co‐infections with SaV and other pathogens were also counted as SaV positive. When two or more cases tested positive for SaV in a mixed infection outbreak, SaV was deemed one of the causal agents.^[^ ^18^ ^]^ Thus, the primary summary data for this study included the number of SaV‐positive AGE cases and the total tested AGE cases (or the positive detection rate), along with the number of SaV‐positive asymptomatic individuals and the total asymptomatic individuals tested. If an article provided such data, two reviewers independently extracted the following details: author, publication year, monitoring province, monitoring periods, monitoring seasons, total tested AGE cases, SaV‐positive AGE cases, total asymptomatic individuals tested, SaV‐positive asymptomatic individuals, detection rate, case category (inpatients or outpatients), gender and age of cases, sample types, and SaV genotypes. The reviewers then input the extracted data into the Epidata 3.0 database and cross‐verified its consistency. In cases of disagreement between the reviewers regarding the data, a third independent reviewer made the final determination.

According to the level of economic development in each province, China is classified into three regions: the high‐income region (encompassing eastern areas of eight provinces, three municipalities, and two special administrative regions–Guangdong, Hainan, Fujian, Taiwan, Zhejiang, Jiangsu, Shandong, and Hebei; Shanghai, Tianjin, and Beijing; Hong Kong and Macao), the middle‐income region (covering central areas of six provinces and the northeastern area of one province–Anhui, Jiangxi, Hunan, Hubei, Henan, Shanxi, and Liaoning), and the low‐income region (including western areas of six provinces, five autonomous regions, a municipality, and northeastern areas of two provinces–Yunnan, Guizhou, Sichuan, Shaanxi, Gansu, and Qinghai; Guangxi, Xizang, Ningxia, Inner Mongolia, and Xinjiang; Chongqing; Jilin and Heilongjiang).^[^ ^19^ ^]^ Traditionally, China is divided into northern and southern regions by the Qinling Mountains‐Huaihe River line.^[^ ^20^ ^]^ The northern region comprises 10 provinces (Hebei, Shandong, Shanxi, Henan, Shaanxi, Gansu, Qinghai, Liaoning, Jilin, and Heilongjiang), four autonomous regions (Inner Mongolia, Xizang, Ningxia, and Xinjiang), and two municipalities (Beijing and Tianjin). The southern region includes the remaining provinces, autonomous regions, and municipalities. China's coastal areas span from north to south and include Liaoning, Hebei, Tianjin, Shandong, Jiangsu, Shanghai, Zhejiang, Fujian, Taiwan, Guangdong, Hong Kong, Macao, Hainan, and Guangxi. The inland region comprises the remaining provinces, autonomous regions, and municipalities. According to the China Information System for Disease Prevention and Control, scattered children refer to those under 3 years old not enrolled in preschool; kindergarten children are aged 3–5.9 and enrolled in preschool; school‐age children are aged 6–13.9 and enrolled in compulsory education; children are defined as aged under 14, and adults as aged 14 and older. In this study, the outbreak setting refers to locations where SaV outbreaks occurred and were treated as a collective entity, including kindergartens, primary schools, middle schools, universities, restaurants, food festivals, villages, and prisons.

### 2.4 Quality Assessment

The quality of routine surveillance studies was assessed using the 2020 Joanna Briggs Institute Critical Appraisal Checklist for prevalence studies.^[^ ^21^ ^]^ Studies with a “yes” score of ≥5 (out of 9) were deemed high quality and included.^[^ ^22^ ^]^ The quality of outbreak studies was evaluated against criteria from previous excellent literature.^[^ ^23^ ^]^ Outbreak studies with a “yes” score of 0 (out of 5) were considered low quality and excluded.^[^ ^23^ ^]^

### 2.5 Statistical Analysis

Heterogeneity across studies was evaluated using the *I* ^2^ test.^[^ ^24^ ^]^ Significant heterogeneity was indicated by *I* ^2^ > 50% and *p* < 0.10, prompting the use of random‐effects models; otherwise, fixed‐effects models were applied.^[^ ^25^ ^]^ Meta‐regression and subgroup analyses were performed to explore heterogeneity sources. When SaV‐positive cases were 0, 0.5 was added for computational purposes.^[^ ^26^ ^]^ Given the non‐normal distribution of data, the Wilcoxon signed‐rank test and Kruskal–Wallis *H* test were used to assess subgroup prevalence differences.^[^ ^23^, ^26^ ^]^ Despite limited data from included articles, SaV genotype data were qualitatively summarized. STATA 12.0 (StataCorp, College Station, TX, USA) was used for data analysis, and R's rworldmap package for mapping. All *p*‐values were two‐tailed, with *p* < 0.10 indicating statistical significance in meta‐regression and *p* < 0.05 in other analyses.

## 3 Results

### 3.1 Literature Search

**Figure 1:**
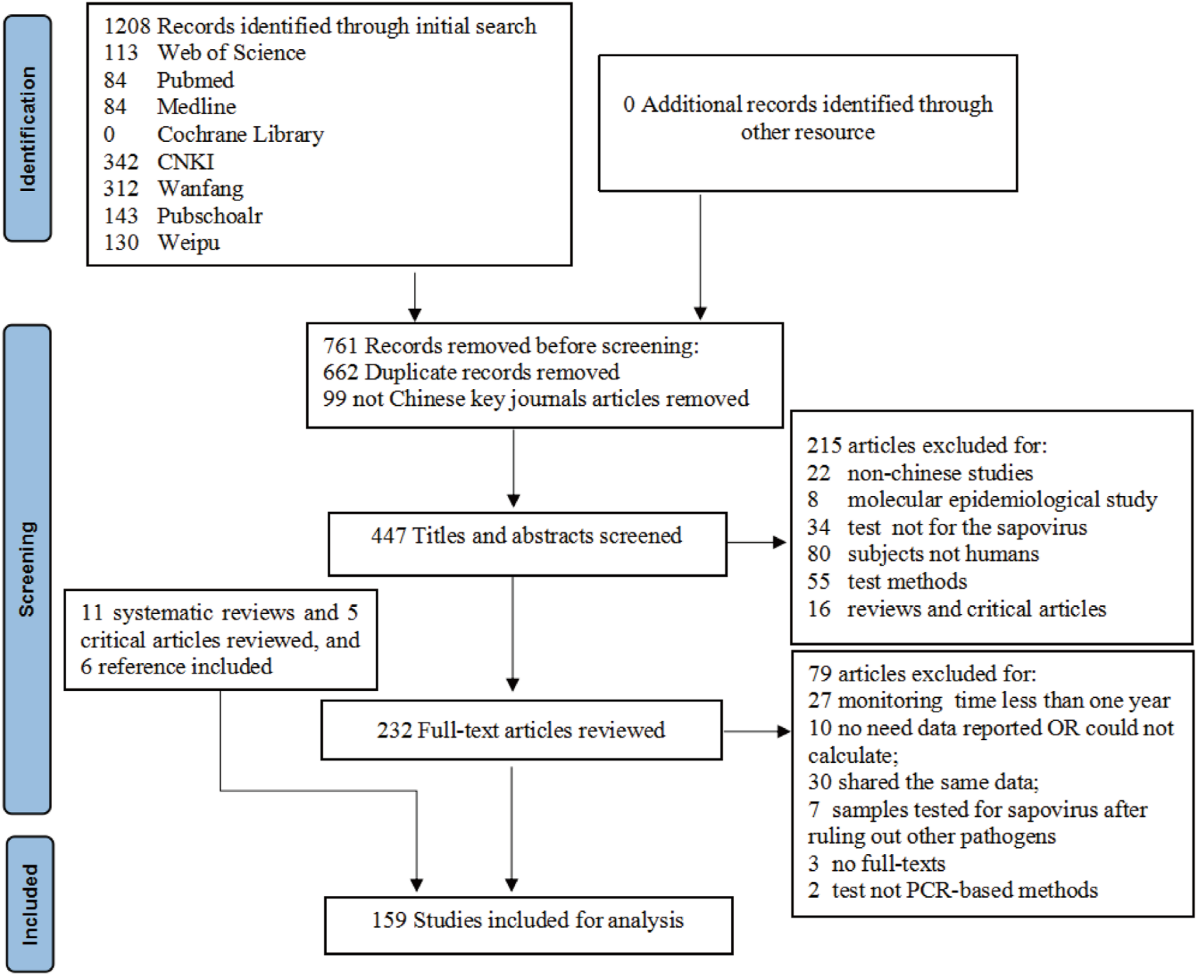
Flow diagram of the article selection process.

The initial literature search retrieved 1208 articles from databases including PubMed, Web of Science, Medline, Cochrane Library, Pubscholar, CNKI, Wanfang, and Weipu. After removing 662 duplicates and excluding 99 articles from non‐Chinese key journals, 215 articles were excluded during title and abstract screening. Full‐text assessment of the remaining 232 articles led to the exclusion of 79 articles based on the inclusion criteria. Meanwhile, manual reference list searching of 11 related systematic reviews, five critical articles, and 153 full‐text readings identified six new articles. According to the study's quality evaluation criteria, 135 routine surveillance articles scored ≥5 points, and 24 outbreak articles were rated as medium or high quality. Ultimately, 159 articles were included in the analysis. Among these, 10 routine surveillance articles covered multiple provinces, six outbreak articles reported multiple outcomes, and some articles presented results across multiple subgroups. This explains why certain subgroup analysis totals do not match the overall total. **Figure** **1** presents a flowchart of the article selection process.

### 3.2 Study Characteristics

**Figure 2:**
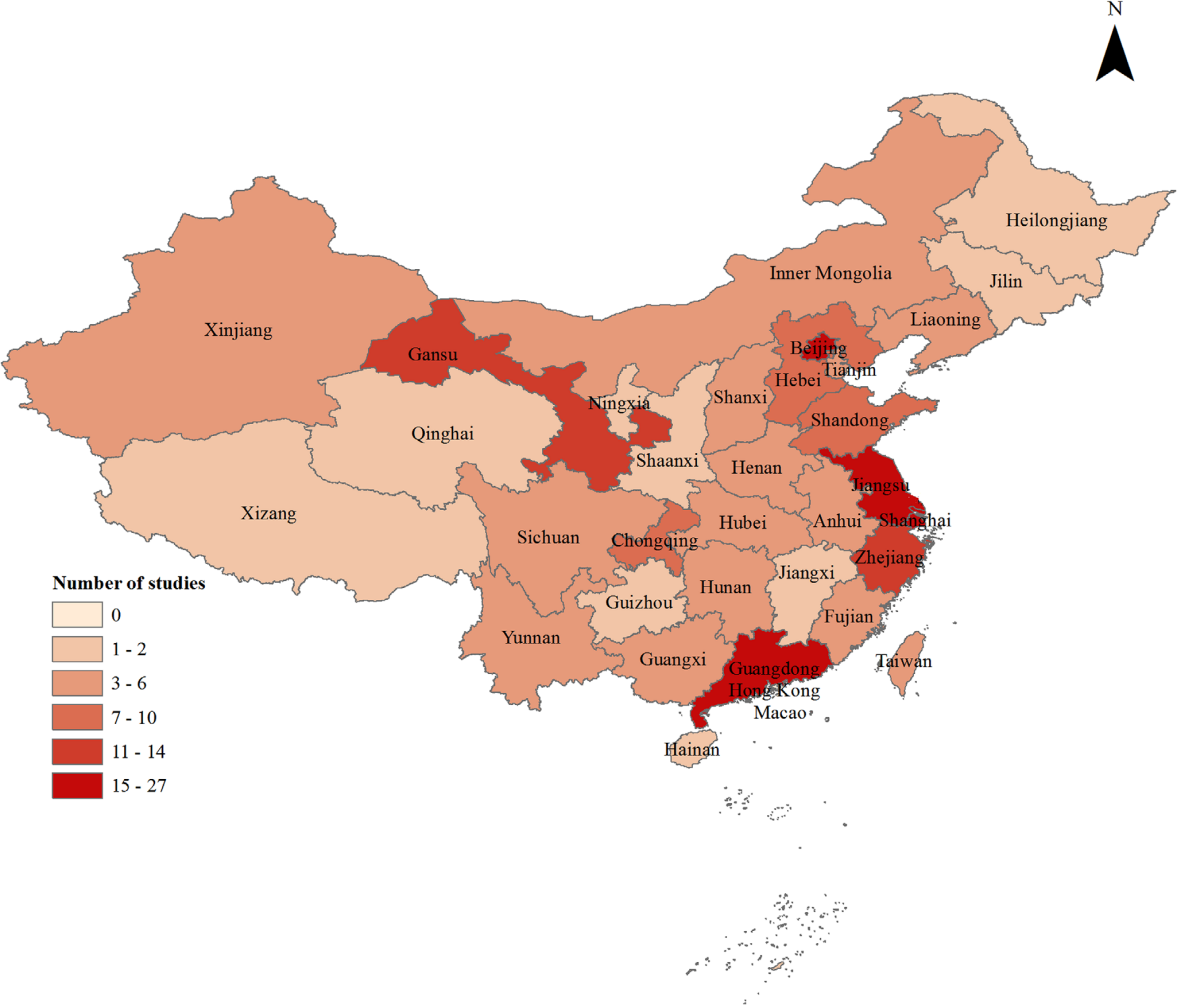
Geographic distribution of articles by province.

A total of 159 articles from 32 Chinese provinces were included, comprising 16 southern and 16 northern provinces, 12 coastal and 20 inland provinces. The highest number focused on Shanghai (27), followed by Guangdong (25), Jiangsu (17), Beijing (15), Zhejiang (14), Gansu (14), Hebei (9), Chongqing (8), Shandong (7), Fujian (6), Henan (6), Hubei (6), Sichuan (6), Hunan (5), Xinjiang (5), Yunnan (5), Anhui (4), Inner Mongolia (4), Guangxi (3), Liaoning (3), Taiwan (3), Tianjin (3), Shanxi (3), Ningxia (2), Qinghai (2), Xizang (2), Jiangxi (2), Hainan (2), Jilin (2), Shaanxi (2), Heilongjiang (2), and Guizhou (1) (**Figure** **2**). High‐income areas contributed 105 articles, middle‐income areas 37, low‐income areas 13, with five unclassifiable articles. The 135 routine surveillance articles covered 207292 AGE patients (interquartile range: 838 [445–1536]), with 4010 SaV‐positive cases. The 24 outbreak articles included 3064 AGE patients, of whom 769 provided fecal or anal swab samples (interquartile range: 18 [11.75–48.25]), with 406 SaV‐positive cases. These studies spanned 2001–2022, with a median surveillance duration of 2 years (range: 1–13). **Tables** **1** and **2** summarize the baseline characteristics and quality assessment findings of the included articles.

**Table 1:** Summary of characteristics of the routine surveillance articles included in the systematic review.

Study	Year	Total number	Positive number	Duration of monitoring [year]	Periods of monitoring	Quality	Province	Per income	Category of cases	Sampling methods	Study design	Case defining symptoms	Sample types	Subject’ group	SaV genome
Kuang Xiaozhou^[1]^	2024	546	5	2	3	6	Shanghai	High	Inpatients	Unknow	1	Vomiting and/or diarrhea	Fecal	3	GII
Wu Qian^[2]^	2024	526	5	3	3	6	Inner Mongolia	Low	Inpatients	Unknow	1	Vomiting and/or diarrhea	Fecal	3	Unknow
Wu Jianmei^[3]^	2024	1348	14	5	2	6	Inner Mongolia	Low	Inpatients	Unknow	1	Diarrhea	Fecal	3	Unknow
Xu Kai^[4]^	2024	1706	34	1.5	3	6	Sichuan	Low	Unknow	Unknow	1	Vomiting and/or diarrhea	Fecal	3,7	Unknow
Ji Xiaolei^[5]^	2024	712	28	2	3	6	Jiangsu	High	Unknow	Unknow	1	Vomiting and/or diarrhea	Fecal	3	GI
Dong Mei^[6]^	2023	392	2	1	3	6	Beijing	High	Unknow	Unknow	1	Diarrhea	Fecal	7	Unknow
Geng Xiayu^[7]^	2023	2914	14	4	1	7	Shanghai	High	Unknow	2	1	Diarrhea	Fecal	6	Unknow
Huang Xianglin^[8]^	2023	2169	56	2	1	6	Shandong	High	Both	Unknow	1	Vomiting and/or diarrhea	Fecal	7	Unknow
Jiao Yang^[9]^	2023	748	21	5	2	6	Beijing	High	Outpatients	Unknow	1	Vomiting and/or diarrhea	Fecal	1,2,3	GI,">GI, GII
Kang Qian^[10]^	2023	2430	26	3	1	6	Gansu	Low	Unknow	Unknow	1	Diarrhea	Fecal	7	Unknow
Li Shuang^[11]^	2023	1522	44	1	1	6	Beijing	High	Outpatients	Unknow	1	Vomiting and/or diarrhea	Fecal	3,7	GI, GII
Liang Yufeng^[12]^	2023	214	8	1	3	6	Guangdong	High	Inpatients	Unknow	1	Diarrhea	Fecal	5	GI
Liu Mingqi^[13]^	2023	763	1	4	2	6	Zhejiang	High	Unknow	Unknow	1	Diarrhea	Fecal	1,2,3	Unknow
Luo Shuhua^[14]^	2023	450	20	4	2	6	Guangdong	High	Unknow	Unknow	1	Diarrhea	Fecal	1,2,3,4,5,6, and 7	Unknow
Shabiremu Tohetamu^[15]^	2023	1173	39	4	2	6	Xinjiang	Low	Inpatients	Unknow	1	Vomiting and/or diarrhea	Fecal	3	Unknow
Wu Bingshan^[16]^	2023	1601	10	6	2	6	Fujian	High	Inpatients	Unknow	1	Diarrhea	Fecal	3	Unknow
Yao Wei^[17]^	2023	797	4	7	2	6	Guangdong	High	Unknow	Unknow	1	Vomiting and/or diarrhea	Fecal	3,7	Unknow
Zhang Qiang^[18]^	2023	1334	36	6	2	6	Anhui	Mid	Inpatients	Unknow	1	Diarrhea	Fecal	3,4,5	Unknow
Gao Qiao^[19]^	2023	1514	11	2	1	7	Shandong	High	Unknow	2	1	Diarrhea	Fecal	7	Unknow
Chen Caiping^[20]^	2022	464	10	3	2	6	Shanghai	High	Outpatients	Unknow	1	Diarrhea	Fecal	6	Unknow
Chen Shuang^[21]^	2022	263	14	4	1	7	Shanghai	High	Outpatients	2	1	Diarrhea	Fecal	5	Unknow
Cheng Ruiduo^[22]^	2022	468	10	4	2	6	Henan	Mid	Unknow	Unknow	1	Diarrhea	Fecal	5	Unknow
Duan Jinqi^[23]^	2022	350	2	2	1	6	Hebei	High	Unknow	Unknow	1	Diarrhea	Fecal	3	Unknow
Han Siyuan^[24]^	2022	982	9	8	1	6	Beijing	High	Outpatients	Unknow	1	Diarrhea	Fecal	6	Unknow
He Fang^[25]^	2022	544	4	4	2	6	Jiangsu	High	Unknow	Unknow	1	Vomiting and/or diarrhea	Fecal/ Anal swabs	7	Unknow
Luo Lingfei^[26]^	2022	1839	74	7	2	7	Shanghai	High	Outpatients	2	1	Diarrhea	Fecal	7	Unknow
Ma Xiaozhen^[27]^	2022	2520	55	10	2	6	Sichuan	Low	Unknow	Unknow	1	Diarrhea	Fecal	3	Unknow
Mao Junwen^[28]^	2022	96	5	2	2	5	Xizang	Low	Outpatients	Unknow	1	Diarrhea	Fecal	1,2,3, and 5	GI, GII
Shabiremu Tohetamu^[29]^	2022	445	36	3	1	6	Xinjiang	Low	Outpatients	Unknow	1	Diarrhea	Fecal	1,2, and 3	Unknow
Yao Shengping^[30]^	2022	316	12	1	2	6	Chongqing	Low	Inpatients	Unknow	1	Diarrhea	Fecal	7	Unknow
Tang Xiang^[31]^	2022	1352	14	3	1	6	Chongqing	Low	Unknow	Unknow	1	Diarrhea	Fecal	1, 2, 3, and 5	GI
Wang Gang^[32]^	2022	5072	209	13	2	6	Chongqing	Low	Unknow	Unknow	1	Diarrhea	Fecal	3	Unknow
Sun Zhenlu^[33]^	2022	713	3	1	1	7	Shandong	High	Unknow	2	1	Diarrhea	Fecal	7	Unknow
Liu Lijuan^[34]^	2022	503	2	1	1	6	Beijing	High	Outpatients	Unknow	1	Vomiting and/or diarrhea	Fecal	6	Unknow
Cao Yihui^[35]^	2021	2262	58	3	1	6	Sichun; Yunnan; Chongqing	Low	Unknow	Unknow	1	Diarrhea	Fecal	1,2,3, and 7	Unknow
Hu Jianying^[36]^	2021	418	28	2	1	6	Yunnan	Low	Unknow	Unknow	1	Diarrhea	Fecal	1,2, and 3	Unknow
Hu Yue^[37]^	2021	1561	14	3	1	6	Chongqing	Low	Outpatients	Unknow	1	Diarrhea	Fecal	1,2,3,4, and 5	GI
Jin Di^[38]^	2021	1347	17	3	1	6	Jiangsu	High	Both	Unknow	1	Vomiting and/or diarrhea	Fecal	3,5,6, and 7	Unknow
Kuang Xiaozhou^[39]^	2021	805	19	3	2	6	Shanghai	High	Inpatients	Unknow	1	Vomiting and/or diarrhea	Fecal	3	Unknow
Li Jing^[40]^	2021	1957	66	3	1	6	Hubei	Mid	Both	Unknow	1	Diarrhea	Fecal	1,3,5,6, and 7	Unknow
Yuan Yan^[41]^	2021	2494	62	8	1	6	Gansu	Low	Both	Unknow	1	Diarrhea	Fecal	3	Unknow
Zhang Weiwei^[42]^	2021	1870	4	6	1	6	Beijing	High	Outpatients	Unknow	1	Diarrhea	Fecal	7	Unknow
Cao Ranran^[43]^	2021	1181	30	5	1	6	Sichuan	Low	Inpatients	Unknow	1	Vomiting and/or diarrhea	Fecal	3	Unknow
Luo Xin^[44]^	2021	656	12	2.5	1	6	Guangdong	High	Outpatients	Unknow	1	Vomiting and/or diarrhea	Fecal	7	GI, GII
Chang Hailing^[45]^	2021	2692	80	4	1	7	Shanghai	High	Outpatients	2	1	Diarrhea	Fecal	1,2, and 3	Unknow
Bi Wenjun^[46]^	2020	1041	38	3.5	1	7	Shanghai	High	Outpatients	2	1	Diarrhea	Fecal	1,5	Unknow
Li Xiaoyu^[47]^	2020	1061	5	1	1	7	Zhejiang	High	Both	1	1	Vomiting and/or diarrhea	Fecal	5	Unknow
Shang Xiaochun^[48]^	2020	1350	57	8	1	6	Zhejiang	High	Outpatients	Unknow	1	Diarrhea	Fecal	1,2, and 3	Unknow
Su Cheng^[49]^	2020	1042	21	3	1	6	Tianjin	High	Unknow	Unknow	1	Vomiting and/or diarrhea	Fecal	7	Unknow
Zhang Wenqiang^[50]^	2020	1397	108	1	1	6	Shandong	High	Both	Unknow	1	Diarrhea	Fecal	7	GI, GII
Wu Limeng^[51]^	2020	6051	153	2	1	7	Shanghai	High	Outpatients	2	1	Diarrhea	Fecal	7	Unknow
Wang Jinxia^[52]^	2020	1216	37	1	1	6	Guangxi	Low	Unknow	Unknow	1	Diarrhea	Fecal	3	Unknow
Zeng Hao^[53]^	2019	1855	66	2	1	6	Hubei	Mid	Unknow	Unknow	1	Diarrhea	Fecal	3,7	Unknow
Gao Lu^[54]^	2019	1536	21	3	1	6	Tianjin	High	Unknow	Unknow	1	Diarrhea	Fecal	7	Unknow
Guan Hongxia^[55]^	2019	3048	10	5	1	6	Jiangsu	High	Outpatients	Unknow	1	Diarrhea	Fecal	7	Unknow
Lin Lin^[56]^	2019	1017	22	6	1	6	Shandong	High	Inpatients	Unknow	1	Diarrhea	Fecal	3	Unknow
Wang Fang^[57]^	2019	219	0	1	1	6	Gansu	Low	Unknow	Unknow	1	Diarrhea	Fecal	3	Unknow
Yuan Jianming^[58]^	2019	428	0	4	1	6	Jiangsu	High	Unknow	Unknow	1	Diarrhea	Fecal	3	Unknow
Zhang Hailong^[59]^	2019	838	9	1	1	6	Guangdong	High	Unknow	Unknow	1	Diarrhea	Fecal	1,6, and 7	GI
Zhang Junying^[60]^	2019	181	4	1	1	6	Beijing	High	Outpatients	Unknow	1	Vomiting and/or diarrhea	Fecal	3	Unknow
Xue Liang^[61]^	2019	569	11	4	1	6	Guangdong	High	Unknow	Unknow	1	Vomiting and/or diarrhea	Fecal	3,7	GI, GII
Chen Chong^[62]^	2019	3060	47	5	1	7	Jiangsu; Zhejiang; Fujian	High	Unknow	2	1	Diarrhea	Fecal	7	Unknow
Cheng Sisi^[63]^	2018	1250	60	6	1	6	Hebei	High	Unknow	Unknow	1	Diarrhea	Fecal	3	Unknow
Gong Xiaohuan^[64]^	2018	9492	194	3	1	7	Shanghai	High	Outpatients	2	1	Diarrhea	Fecal	7	Unknow
Gong Chunhua^[65]^	2018	617	13	2	1	6	Shanghai	High	Outpatients	Unknow	1	Diarrhea	Fecal	5	Unknow
Guo Limin^[66]^	2018	463	6	3	1	6	Henan	Mid	Unknow	Unknow	1	Diarrhea	Fecal	Unable to group	Unknow
Jiang Xiao^[67]^	2018	300	7	2	1	6	Jiangsu	High	Outpatients	Unknow	1	Vomiting and/or diarrhea	Fecal/ Anal swabs	5	Unknow
Jin Jing^[68]^	2018	3009	0	1	1	6	Hubei	Mid	Both	Unknow	1	Diarrhea	Fecal	3	Unknow
Luo Kaiwei^[69]^	2018	1123	17	2	1	6	Hunan	Mid	Unknow	Unknow	1	Diarrhea	Fecal	1,3, and 7	Unknow
Tan Weiwei^[70]^	2018	363	3	1	1	6	Jiangsu	High	Outpatients	Unknow	1	Diarrhea	Fecal	7	Unknow
Zhang Yalin^[71]^	2018	303	13	1	1	6	Hubei	Mid	Unknow	Unknow	1	Diarrhea	Fecal	3,7	Unknow
He Liying^[72]^	2017	850	3	1	1	6	Yunnan	Low	Unknow	Unknow	2	Diarrhea	Fecal	3	Unknow
Su Tong^[73]^	2017	2211	52	6	1	6	Hebei	High	Inpatients	Unknow	1	Diarrhea	Fecal	3	Unknow
Sun Jingshuang^[74]^	2017	201	5	2	1	7	Shanghai	High	Outpatients	2	1	Diarrhea	Fecal	7	Unknow
Wang Haiyan^[75]^	2017	671	12	1	1	6	Shandong	High	Outpatients	Unknow	1	Vomiting and/or diarrhea	Fecal	7	Unknow
Xie Sirou^[76]^	2017	600	14	1.5	1	6	Guangdong	High	Unknow	Unknow	1	Diarrhea	Fecal	3,7	Unknow
Yuan Lu^[77]^	2017	400	10	1	1	6	Qinghai	Low	Outpatients	Unknow	1	Diarrhea	Fecal	1,2, and 3	Unknow
Zhou Ying^[78]^	2017	1754	95	4	1	6	Beijing	High	Outpatients	Unknow	1	Vomiting and/or diarrhea	Fecal	3	Unknow
Chen Wanbing^[79]^	2016	511	24	1	1	6	Chongqing	Low	Unknow	Unknow	1	Diarrhea	Fecal	1,2, and 3	GI, GII
Ding Ming^[80]^	2016	4214	69	2	1	6	Shanghai	High	Outpatients	Unknow	1	Diarrhea	Fecal	7	Unknow
Pang Beibei^[81]^	2016	1368	105	2	1	6	Hubei	Mid	Unknow	Unknow	1	Vomiting and/or diarrhea	Fecal	1,3,4,5,6, and 7	GI, GII
Su Jing^[82]^	2016	987	6	3	1	6	Jiangsu	High	Outpatients	Unknow	1	Diarrhea	Fecal	7	Unknow
Wang Dongyue^[83]^	2016	2462	13	4	1	6	Jiangsu	High	Unknow	Unknow	1	Diarrhea	Anal swabs	7	Unknow
Xu Zhongqing^[84]^	2016	1554	40	2.5	1	6	Shanghai	High	Unknow	Unknow	1	Vomiting and/or diarrhea	Fecal	6	Unknow
Zhao Jiayong^[85]^	2016	2541	51	8	1	6	Henan	Mid	Unknow	Unknow	1	Diarrhea	Fecal	3	Unknow
Zheng Shufa^[86]^	2016	9364	100	5	1	6	Zhejiang	High	Outpatients	Unknow	1	Diarrhea	Fecal	5,6, and 7	Unknow
Zhou Yongkang^[87]^	2016	3175	22	3	1	6	1	High; Low; Mid	Inpatients	Unknow	1	Diarrhea	Fecal	1,2, and 3	GI, GII
Shen Hongwei^[88]^	2016	412	9	1	1	6	Guangdong	High	Outpatients	Unknow	1	Diarrhea	Fecal	3,7	Unknow
Zhang ShunXian^[89]^	2016	1121	3	1	1	6	Yunnan	Low	Outpatients	Unknow	2	Diarrhea	Fecal	1,3, and 7	GI, GII
Li L L^[90]^	2016	461	30	2	1	6	Hebei; Hunan	High; Mid	Inpatients	Unknow	2	Diarrhea	Fecal	3	Unknow
Dong Hongyan^[91]^	2015	336	7	1	1	6	Jiangsu	High	Unknow	Unknow	1	Diarrhea	Fecal	7	Unknow
Hong Zhantong^[92]^	2015	1587	11	3	1	6	Guangdong	High	Both	Unknow	1	Diarrhea	Fecal	7	Unknow
Jin Miao^[93]^	2015	519	19	1	1	6	Beijing	High	Outpatients	Unknow	1	Vomiting and/or diarrhea	Fecal	6	GI, GII
Luo Xin^[94]^	2015	273	4	1	1	6	Guangdong	High	Both	Unknow	1	Diarrhea	Fecal	7	GI
Xie Jing^[95]^	2015	232	8	1	1	6	Gansu	Low	Unknow	Unknow	1	Diarrhea	Fecal	3	GI, GII
Yu Jianxing^[96]^	2015	41 553	467	5	1	6	2	High; Low; Mid	Unknow	Unknow	1	Diarrhea	Fecal	7	Unknow
Zhang Min^[97]^	2015	532	10	1	1	6	Zhejiang	High	Outpatients	Unknow	1	Diarrhea	Fecal	7	Unknow
Zhang Yonghong^[98]^	2015	286	14	1	1	6	Ningxia	Low	Both	Unknow	1	Diarrhea	Fecal	1,2,3,4, and 5	Unknow
Zhang D‐M^[99]^	2015	1128	14	2.5	1	6	Guangdong	High	Both	Unknow	1	Diarrhea	Fecal	5	Unknow
Lu Lijuan^[100]^	2015	436	2	2	1	6	Shanghai	High	Outpatients	Unknow	1	Vomiting and/or diarrhea	Fecal	3	GI, GII
Li Ke^[101]^	2015	508	5	2	1	6	Hebei; Hunan	High; Mid	Inpatients	Unknow	2	Vomiting and/or diarrhea	Fecal	3	Unknow
Chen Huifang^[102]^	2014	709	9	2	1	7	Guangdong	High	Outpatients	3	1	Diarrhea	Fecal	3	Unknow
Hong Wansheng^[103]^	2014	507	12	1	1	6	Zhejiang	High	Outpatients	Unknow	1	Diarrhea	Fecal	1,2,3, and 7	Unknow
Li Ruiqiang^[104]^	2014	282	8	1	1	7	Beijing	High	Outpatients	1	1	Diarrhea	Fecal	6	Unknow
Qiao Kun^[105]^	2014	539	7	1	1	7	Shanghai	High	Unknow	2	1	Diarrhea	Fecal	6	Unknow
Xiang Jingyao^[106]^	2014	331	10	1	1	6	Gansu	Low	Inpatients	Unknow	1	Vomiting and/or diarrhea	Fecal	3	GI, GII
Zhao Jin^[107]^	2014	2416	65	4	1	6	Chongqing	Low	Outpatients	Unknow	1	Diarrhea	Fecal	3,7	Unknow
Wu Wei^[108]^	2014	983	15	1	1	6	Guangdong	High	Outpatients	Unknow	1	Vomiting and/or diarrhea	Fecal	7	GI, GII
Wang Gang^[109]^	2014	1125	42	2	1	6	Shanghai	High	Outpatients	Unknow	1	Vomiting and/or diarrhea	Fecal	6	GI, GII
An Shuyi^[110]^	2013	639	2	3	1	6	Liaoning	Mid	Unknow	Unknow	1	Diarrhea	Fecal	7	Unknow
Fei Yi^[111]^	2013	619	17	1	1	6	Shanghai	High	Unknow	Unknow	1	Diarrhea	Fecal	3	GI, GII
Hu Tingting^[112]^	2013	487	9	1	1	6	Guangdong	High	Unknow	Unknow	1	Vomiting and/or diarrhea	Fecal	1,2,3,6, and 7	GI, GII
Lu Lijuan^[113]^	2013	1110	2	6	1	6	Shanghai	High	Both	Unknow	1	Diarrhea	Fecal	3	GI, GII
Miu Guozhong^[114]^	2013	66	1	1	1	5	Jiangsu	High	Outpatients	Unknow	1	Diarrhea	Fecal	7	Unknow
Shabiremu Tohetamu^[115]^	2013	379	4	1	1	6	Xinjiang	Low	Inpatients	Unknow	1	Diarrhea	Fecal	1,3	Unknow
Ye Hongyan^[116]^	2013	900	44	1	1	6	Zhejiang	High	Outpatients	Unknow	1	Diarrhea	Fecal	3	Unknow
Chen Shih‐Yen^[117]^	2013	755	6	4.5	1	6	Taiwan	High	Inpatients	Unknow	1	Vomiting and/or diarrhea	Fecal	5	Unknow
Chen Yu^[118]^	2013	811	36	1	1	7	Jiangsu; Zhejiang; Fujian	High	Outpatients	1	1	Diarrhea	Fecal	3	Unknow
Li Xiaole^[119]^	2012	428	5	1	1	6	Jiangsu	High	Both	Unknow	2	Diarrhea	Fecal	3	GI, GII
Li Xiaoyi^[120]^	2012	414	11	1	1	6	Hebei	High	Inpatients	Unknow	1	Diarrhea	Fecal	3	Unknow
Lin Qian^[121]^	2012	300	4	1	1	6	Jiangsu	High	Unknow	Unknow	1	Diarrhea	Fecal	3	Unknow
Wang Yongxia^[122]^	2012	295	12	1	1	6	Gansu	Low	Both	Unknow	1	Diarrhea	Fecal	3	GI, GII
Liu Zhihua^[123]^	2011	985	10	1	1	6	Guangdong	High	Outpatients	Unknow	1	Diarrhea	Fecal	3	GI, GII
Tan Dongmei^[124]^	2011	412	19	2	1	6	Guangxi	Low	Outpatients	Unknow	1	Vomiting and/or diarrhea	Fecal	6	Unknow
Wang Yange^[125]^	2011	852	16	1	1	6	Guangdong	High	Unknow	Unknow	1	Vomiting and/or diarrhea	Fecal	1,2,3,5,6, and 7	GI, GII
ZhuGe Xiaoling^[126]^	2011	920	11	1	1	7	Zhejiang	High	Outpatients	1	1	Diarrhea	Fecal	3, 5, 6, and 7	GI, GII
ZhuGe Xiaoling^[127]^	2011	2144	130	2	1	7	Zhejiang	High	Outpatients	1	1	Vomiting and/or diarrhea	Fecal	1,3,5,6, and 7	GI, GII
Gong Zhixiang^[128]^	2010	910	59	1	1	7	Shanghai	High	Outpatients	2	1	Vomiting and/or diarrhea	Fecal	5	GI, GII
Li Bowen^[129]^	2010	345	5	1	1	6	Gansu	Low	Inpatients	Unknow	2	Diarrhea	Fecal	3	GI, GII
Li Yongqing^[130]^	2010	2118	28	1	1	6	Gansu	Low	Inpatients	Unknow	1	Diarrhea	Fecal	3	GI, GII
Chang Zhaorui^[131]^	2009	1100	10	1	1	6	3	High; Low; Mid	Inpatients	Unknow	1	Diarrhea	Fecal	3	GI, GII
Jin Yu^[132]^	2009	544	6	2	1	6	Gansu	Low	Inpatients	Unknow	1	Diarrhea	Fecal	3	GI, GII
Cheng Weixia^[133]^	2008	286	5	1	1	6	Gansu	Low	Inpatients	Unknow	2	Diarrhea	Fecal	3	GI, GII
Ye Xinhua^[134]^	2008	1062	12	5	1	6	Gansu	Low	Inpatients	Unknow	1	Diarrhea	Fecal	3	GI, GII
Sun Yaping^[135]^	2006	414	5	1	1	6	Fujian; Gansu; Henan	High; Low; Mid	Inpatients	Unknow	1	Diarrhea	Fecal	3	GI, GII

[ ]: included reference numbers in the “Routine surveillance studies” content in Additional File S1 (Supporting Information).

Total number: the number of cases tested in every study.

Periods of monitoring: 1) Before the COVID‐19 pandemic (Before 2020); 2) Including COVID‐19 pandemic and non‐epidemic period; 3) During the COVID‐19 pandemic (2020–2022)

Province: 1) Inner Mongolia, Hunan, Sichuan, Shanxi, Xinjiang, Shanghai, Heilongjiang, Hebei; 2) Anhui, Beijing, Fujian, Gansu, Guangdong, Guangxi, Guizhou, Hainan, Hebei, Henan, Heilongjiang, Hubei, Hunan, Jilin, Jiangsu, Jiangxi, Liaoning, Inner Mongolia, Ningxia, Qinghai, Shandong, Shanxi, Shaanxi, Shanghai, Sichuan, Tianjin, Xizang, Xinjiang, Yunan, Zhejiang, Chongqing; 3) Anhui, Fujian, Hainan, Hebei, Henan, Jilin, Shanxi, Shaanxi, Shanghai

Sampling methods: 1) Simple random sampling; 2) Systematic sampling; 3)Chester sampling

Study design: 1) Cross‐sectional study; 2) Case‐control study

Subject'group: 1) <3 years old; 2) 3–5.9 years old; 3)<6 years old; 4) 6–13.9 years old; 5)<14 years old; 6) Adults; 7) Entire population

Quality: JBI critical appraisal list for studies reporting prevalence data: (1) was the sample frame appropriate to address the target population? (2) were study participants sampled in an appropriate way? (3) was the sample size adequate? (4) were the study subjects and the setting described in detail? (5) was the data analysis conducted with sufficient coverage of the identified sample? (6) were valid methods used for the identification of the condition? (7) was the condition measured in a standard, reliable way for all participants? (8) was there an appropriate statistical analysis? (9) was the response rate adequate, and if not, was the low response rate managed appropriately? The aggregate score was 9. One point was added for each “yes” answer, and no point was added for each “no” or “unclear” answer. From: Munn Z, Moola S, Lisy K, Riitano D, Tufanaru C. Chapter 5: Systematic reviews of prevalence and incidence. In: Aromataris E, Munn Z (Editors). JBI Manual for Evidence Synthesis. JBI, 2020. Available online: https://jbi.global/critical‐appraisal‐tools.

**Table 2:** Characteristics of outbreak‐related articles in the systematic review.

Study	Year	Number of case samples	Positive number	Number of exposed individuals	Number of cases	Quality	Per income	Province	Outbreak setting	Subject'group	Sample types	NoV gentypes	Case definition
Chen Xiaobin	2024	43	20	1961	148	5	High	Guangdong	Middle school	Adults	Anal swab	GI.6	Y
Tian Jun^[2]^	2023	46	22	4056	46	3	Mid	Liaoning	College	Adults	Anal swab	GI.6	Y
Shi Rongjie^[3]^	2023	26	3	2256	26	5	High	Guangdong	High school	Adults	Anal swab, Hand swab	unknown	Y
Yao Ping^[4]^	2023	50	28	8004	118	2	High	Jiangsu	Kindergarten, Primary school	Children	Anal swab	GII.3, GI.1, GI.3, GII.2	Y
Wang Peng^[5]^	2023	17	11	72	34	3	Low	Gansu	Kindergarten	Children	Anal swab	GI.2	N
Su Lingxuan^[6]^	2023	105	60	unknown	105	2	High	Zhejiang	Primary school, Kindergarten, High school	Children	Fecal	GI.6, GII.5, GI.2, GI.1	Y
Zou Lin^[7]^	2023	83	39	1588	996	5	High	Beijing	High school	Adults	Fecal, Anal swab	GI	N
Feng Taicong^[8]^	2022	107	39	5068	120	3	High	Shanghai	Primary school, Kindergarten	Children	Anal swab	unknown	Y
Li Dongliang^[9]^	2022	14	5	2144	66	5	Mid	Anhui	college	Adults	Anal swab	unknown	Y
Zhu Lin^[10]^	2021	6	5	27	12	5	High	Shanghai	Kindergarten	Children	Anal swab	GI.1	Y
Wu FangTzy^[11]^	2021	16	13	unknown	33	3	High	Taiwan	Kindergarten, Primary school, Prison	Children,Adults	Fecal	GI.1, GII.3, GII.8	N
Li Janyi^[12]^	2020	49	19	3531	422	5	High	Guangdong	Community Village	Entire population	Fecal, Anal swab	unknown	Y
Li Yang^[13]^	2020	9	5	65	13	4	High	Beijing	Kindergarten	Children	Anal swab	unknown	Y
Guo Li^[14]^	2020	56	34	301	78	3	High	Beijing	Primary school, Kindergarten	Children	Anal swab, Fecal,Vomitus	unknown	Y
Yan Yuxiao^[15]^	2020	11	7	1945	482	5	High	Guangdong	Primary school	Children	Fecal	GII.8	Y
Huang Siyue	2020	14	6	11800	90	5	Mid	Jiangxi	college	Adults	Anal swab, Fecal,Vomitus	unknown	Y
Huang YanFei^[17]^	2019	24	23	53	26	5	High	Guangdong	Primary school	Children, Adults	Anal swab	unknown	Y
Xing Yan^[18]^	2018	14	9	33	14	5	High	Beijing	Kindergarten	Children, Adults	Fecal	unknown	Y
Wang Jinjin^[19]^	2018	19	15	77	29	4	High	Guangdong	Kindergarten	Children	Fecal, Anal swab	GII.3, GI.2	Y
Long DongLing^[20]^	2017	14	11	unknown	14	3	High	Guangdong	Kindergarten	Children, Adults	Fecal	GII.3	N
Wang Yan^[21]^	2015	25	15	unknown	78	4	High	Shanghai	Food festival exhibition	Adults	Fecal	GI.2, GI.3	Y
Xu Chunhua^[22]^	2014	6	6	337	32	4	High	Shanghai	Primary school	Children, Adults	Fecal	unknown	Y
He Yaqing^[23]^	2012	7	4	200	27	3	High	Guangdong	Restaurant	Adults	Fecal	GI.2	Y
Wu FangTzy^[24]^	2008	8	7	unknown	55	3	High	Taiwan	College	Adults	Fecal	GI.2	N

[ ]: included reference numbers in the “Outbreak Studies” content in Additional File S1 (Supporting Information). Case definition: Y = Clear case definition provided; N = No clear case definition provided.

Quality: Critical appraisal list for outbreak studies reporting prevalence data: 1) clearly described the time, place, and population of the outbreak; 2) clearly described pathogen detection, field hygienic investigation, and interventions; 3) described the investigation of risk factors for outbreaks; 4) conducted scientific statistical tests; 5) used randomization, stratification, and/or pairing for bias controlling, or discussed any potential confounding factors. The aggregate score was 5. One Scoring: 1 point for each “yes” answer, 0 for “no” or “unclear.” Total score: 5. Quality rating: 3–5 = High, 1–2 = Moderate, 0 = Low. From: Ding Z, Zhai Y, Wu C, Wu H, Lu Q, Lin J, He F. Infectious diarrheal disease caused by contaminated well water in Chinese schools: A systematic review and meta‐analysis. J Epidemiol. 2017 Jun;27(6):274–281. https://doi.org/10.1016/j.je.2016.07.006.

Of the 135 routine surveillance studies, 128 were cross‐sectional, and seven were case‐control studies; no cohort studies were conducted. Among the 99 articles focusing on AGE cases, only diarrhea was explicitly mentioned in some without reference to vomiting, while 36 articles highlighted both diarrhea and/or vomiting. By age group, 26 studies focused on scattered children, 18 on kindergarten children, five on school‐age children, 23 on children, 20 on adults, and 54 covered the entire population. Regarding case category, 27 studies involved hospitalized cases, 49 outpatient cases, 13 both, and 47 were not detailed. For surveillance periods, 112 studies predated the COVID‐19 pandemic, six occurred during it, and 17 spanned both. By per capita income, 85 studies were in high‐income provinces, 12 in middle‐income, 34 in low‐income, with five studies not specifying. Geographically, 85 studies were in southern provinces, 46 in northern, with five studies not detailed; 78 in coastal, 54 in inland, with four not detailed. Gender distribution was reported in 32 articles, seasonal in 30, and the infection status of healthy controls in seven.

### 3.3 Pooled Prevalence of SaV

**Figure 3:**
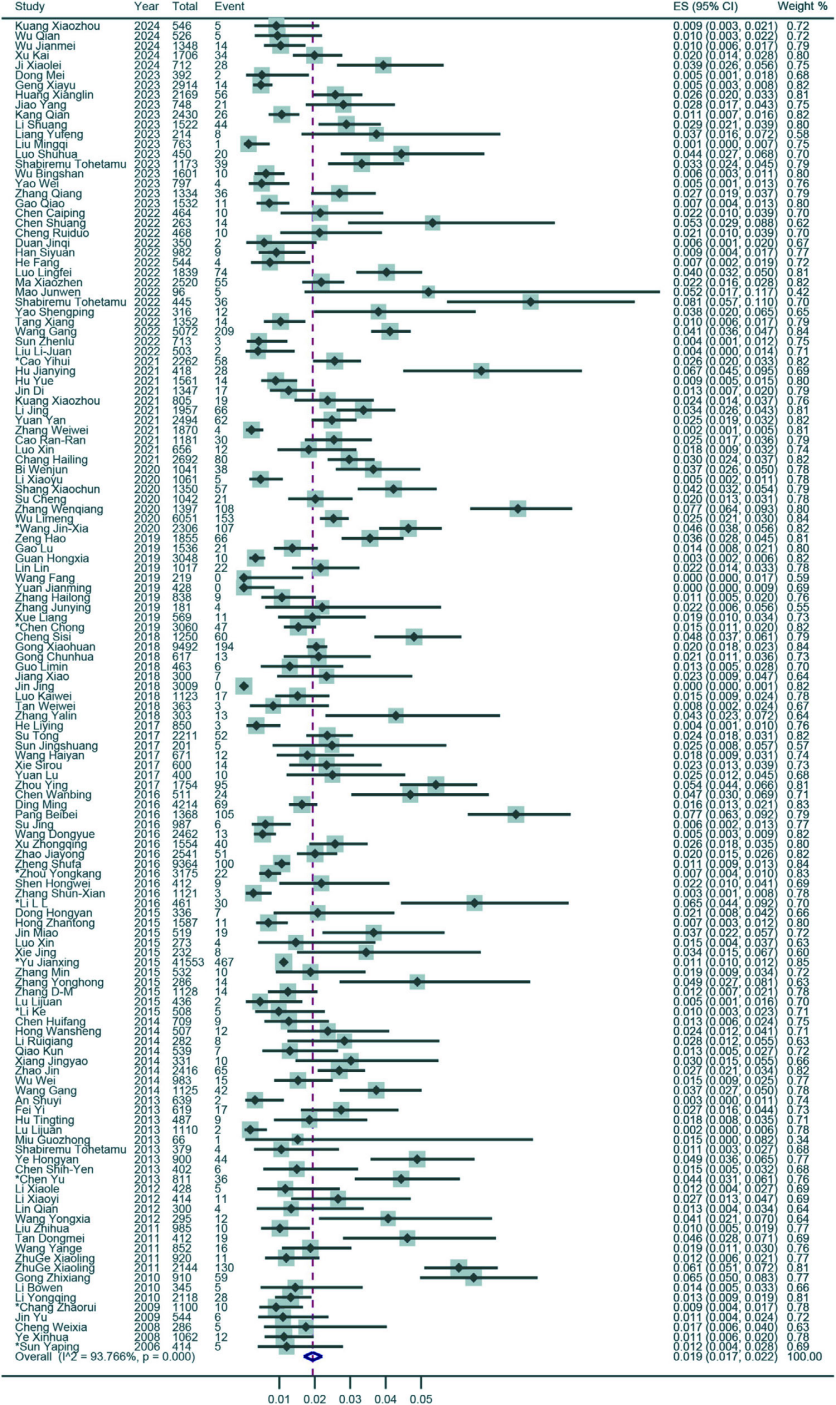
Forest plot of prevalence estimates from routine surveillance articles. Results of 135 routine surveillance articles estimating the prevalence of SaV infection. Event: Number of SaV‐positive AGE cases; Total: Number of AGE cases whose samples were tested; *: Studies with prevalence were calculated in *N* provinces (*N* > 1).

The prevalence of SaV in sporadic AGE cases was determined to be 1.9% (95% CI: 1.7–2.2; *I* ^2^ = 93.61%, *τ* ^2^ = 0.010, *p* < 0.001 for heterogeneity) across 135 studies spanning 32 Chinese provinces (**Figure** **3**). After excluding three studies with non‐fecal samples, the prevalence remained consistent at 1.9% (95% CI: 1.7–2.2; *I* ^2^ = 93.64%, *τ* ^2^ = 0.010, *p* < 0.001 for heterogeneity) in 132 studies using fecal samples. Among 1836 healthy controls from seven studies, the asymptomatic SaV prevalence was significantly lower at 0.8% (95% CI: 0–2.5; *I* ^2^ = 87.10%, *τ* ^2^ = 0.027, *p* < 0.001 for heterogeneity) (Figure S1, Supporting Information).

**Figure 4:**
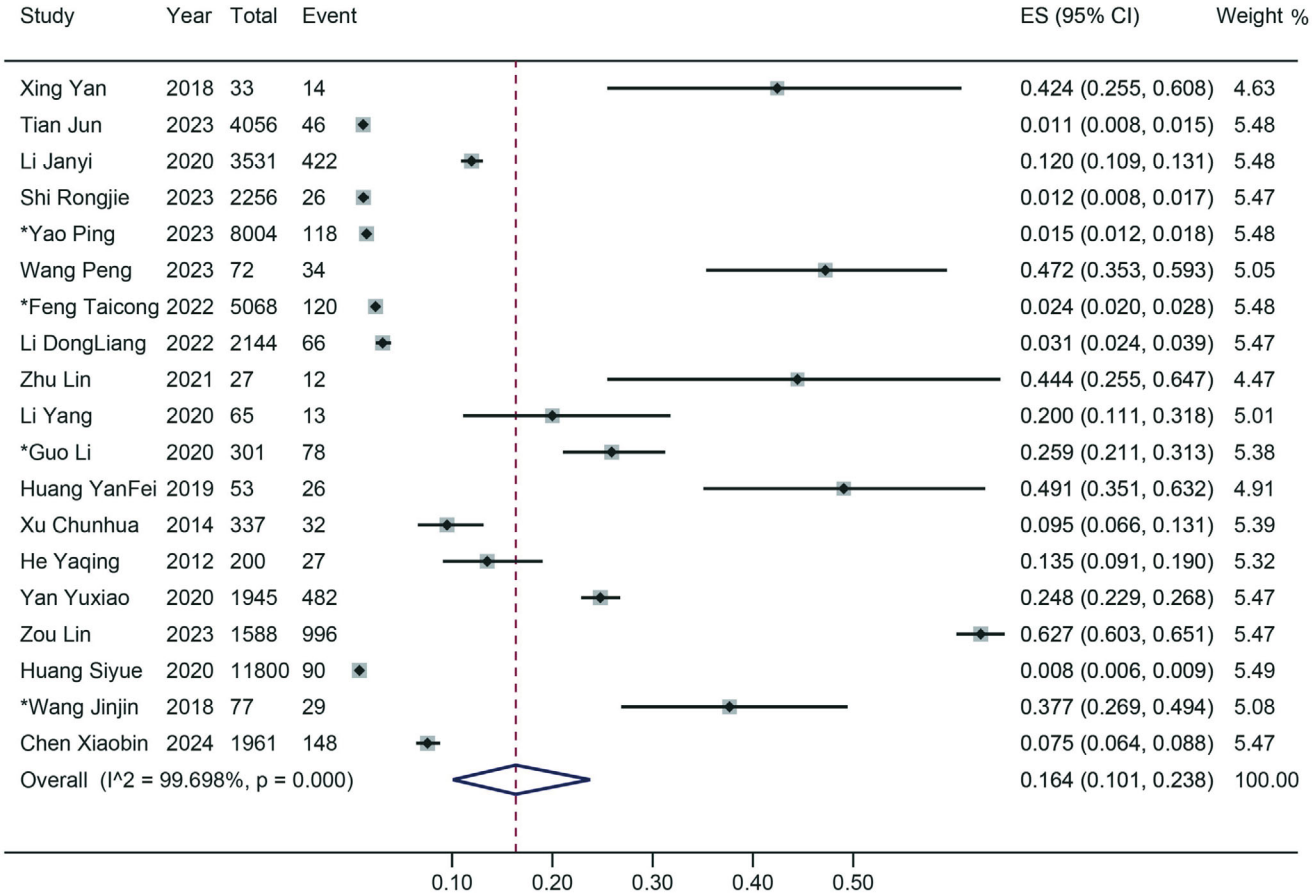
Forest plot of prevalence estimates from outbreak articles. Results of 19 outbreak articles estimating the prevalence of SaV Infection. Event: Number of AGE cases; Total: Number of individuals exposed to SaV in outbreaks; *: Studies with prevalence were calculated in *N* outbreaks (*N* > 1).

In the 24 outbreak studies, the pooled SaV detection rate was substantially higher at 58.8% (95% CI: 50.4–67; *I* ^2^ = 77.09%, *τ* ^2^ = 0.107, *p* < 0.001 for heterogeneity) (Figure S2, Supporting Information). A separate analysis of 19 outbreak studies estimated the SaV prevalence to be 16.4% (95% CI: 10.1–23.8; *I* ^2^ = 99.70%, *τ* ^2^ = 0.160, *p* < 0.001 for heterogeneity) (**Figure** **4**). Notably, when applying a fixed‐effect model to data from 11 outbreak studies, the asymptomatic SaV prevalence was 14.4% (95% CI: 8.9–20.7; *I* ^2^ = 0%, *p* = 0.999 for heterogeneity), indicating no heterogeneity in these studies (Figure S3, Supporting Information).

### 3.4 Meta‐Regression Analysis

The sources of heterogeneity were explored via univariate meta‐regression across 135 routine surveillance studies. Eight subgroups were examined: geographic region (South/North and Coastal/Inland), case category, monitoring period, monitoring season, case's gender, case's age, and per capita income. Among these, only the case's age demonstrated statistical significance in the meta‐regression analysis (*p* = 0.091). Notably, due to some articles reporting results across multiple subgroups, a multivariate meta‐regression analysis was not feasible. Furthermore, meta‐regression results indicated that the publication year of the studies had no significant impact on the prevalence of SaV in AGE cases (*p* = 0.371).

### 3.5 Subgroup Analysis of Prevalence

**Figure 5:**
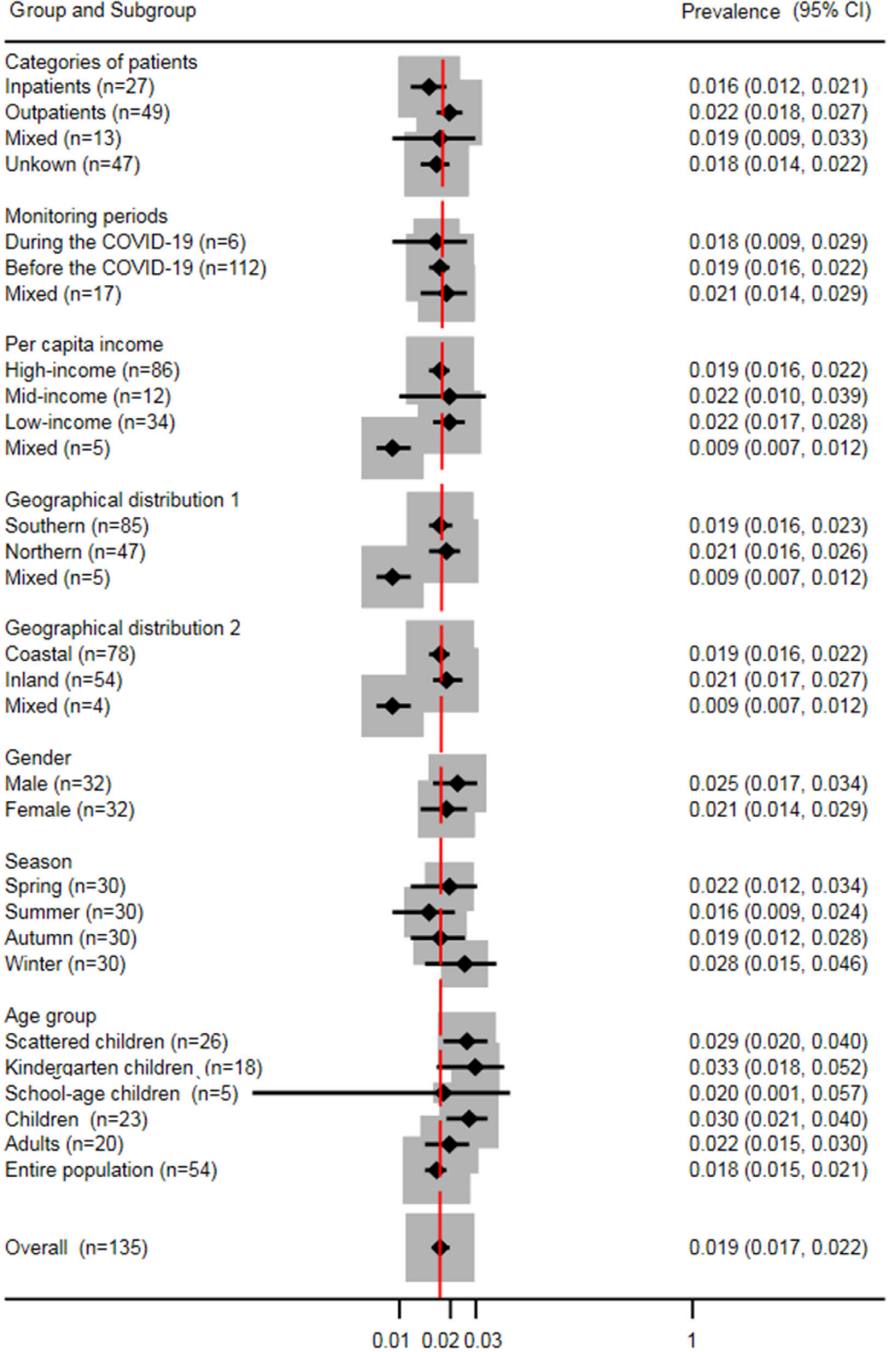
Subgroup pooled prevalence estimates of SaV infection. *N*: Number of articles. Line segment length: Confidence interval of the pooled estimate. Black polygon: Estimated point prevalence for each subgroup. Red dashed lines: Overall summary estimates for comparison. For all subgroups, *P*‐values for heterogeneity tests were *P* < 0.05, indicating significant heterogeneity. Despite stratification by various factors, significant heterogeneity persists within each subgroup, highlighting the complexity of prevalence estimates across different study settings.

The prevalence results for the eight prespecified factor subgroups are summarized in **Figure** **5**. The analysis identified significant heterogeneity within specific subgroups of each grouping factor (*p* < 0.05). Therefore, subgroup analysis alone was insufficient to pinpoint the exact sources of this heterogeneity. The detailed subgroup analysis results are as follows: When stratified by geographic location (South vs North), the prevalence of SaV was comparable between northern China (2.1%, 95% CI 1.6–2.6) and southern China (1.9%, 95% CI 1.6–2.3) (*p* = 0.821). Similarly, when categorized by coastal versus inland regions, the prevalence rates were similar in coastal provinces (1.9%, 95% CI 1.6–2.2) and inland provinces (2.1%, 95% CI 1.7–2.7) (*p* = 0.240). Regarding per capita income, middle‐income areas (2.2%, 95% CI 1–3.9) and low‐income areas (2.2%, 95% CI 1.7–2.8) showed similar SaV prevalence rates, both of which were slightly higher than high‐income regions (1.9%, 95% CI 1.6–2.2) (*p* = 0.329). Seasonal analysis revealed the highest SaV prevalence in winter (2.8%, 95% CI 1.5–4.6), followed by spring (2.2%, 95% CI 1.2–3.4), autumn (1.9%, 95% CI 1.2–2.8), and summer (1.6%, 95% CI 0.9–2.4) (*p* = 0.656). In terms of case category, outpatient cases exhibited a slightly higher SaV prevalence (2.2%, 95% CI 1.8–2.7) compared to inpatient cases (1.6%, 95% CI 1.2–2.1) (*p* = 0.133). Gender‐based analysis showed a slightly higher prevalence in males (2.5%, 95% CI 1.7–3.4) than in females (2.1%, 95% CI 1.4–2.9) (*p* = 0.207). Temporal analysis comparing pre‐pandemic and pandemic periods revealed a marginally higher prevalence before the COVID‐19 pandemic (1.9%, 95% CI 1.6–2.2) than during the pandemic (1.8%, 95% CI 0.9–2.9) (*p* = 0.759). Age‐stratified analysis demonstrated an upward trend in SaV prevalence among scattered children (2.9%, 95% CI 2–4), peaking in kindergarten children (3.3%, 95% CI 1.8–5.2). This was followed by a decline in school‐age children (2%, 95% CI 0.1–5.7) and a second peak in adults (2.2%, 95% CI 1.5–3). However, these age‐related differences did not reach statistical significance (*p* = 0.215).

### 3.6 Association between SaV and AGE

**Figure 6:**
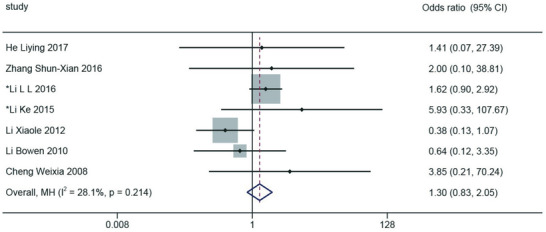
Forest plot of odds ratios for SaV infection and AGE symptoms based on case‐control studies.

In seven routine surveillance studies that reported the infection status of healthy control individuals, our meta‐analysis using a fixed‐effect model revealed no significant association between SaV infection and AGE, with an odds ratio (OR) of 1.30 (95% confidence interval [CI]: 0.83–2.05), *I* ^2^ = 28.1%, *p* = 0.214, and heterogeneity = 1.180, as shown in **Figure** **6**.

### 3.7 Genetic Diversity of SaV

Among the sporadic cases reported as SaV‐positive across the 135 routine surveillance studies, a limited subset of cases (12.07%, 484/4010) underwent further genotyping analysis. Of these genotyped cases, 66.12% (320/484) were identified as GI genome, 17.98% (87/484) as GII genome, and 8.88% (43/484) as GIV genome. The remaining cases (7.02%, 34/484) could not be successfully genotyped. Detailed subtype information was available for 64.26% (311/484) of GI genome cases and 17.98% (87/484) of GII genome cases. The three most prevalent SaV genotypes identified were GI.1 (35.54%, 172/484), GI.2 (24.59%, 119/484), and GII.1 (11.98%, 58/484), highlighting the predominance of GI strains in the study population.

**Figure 7:**
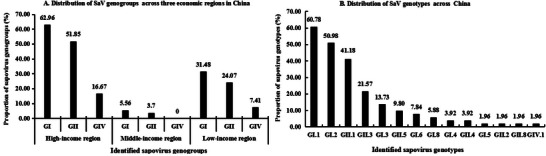
Genetic distribution of sapovirus strains identified in China.

Among the 159 included articles, 66.04% (105/159) reported SaV infections without providing genome information, 1.89% (3/159) included SaV genome information without further genotyping, and the remaining 32.08% (51/159) detailed SaV genotype information. Within these 54 articles, GI genomes were reported more frequently (72.22%, 39/54) compared to GII (62.96%, 34/54) and GIV genomes (24.07%, 13/54). This trend was consistent across high‐, middle‐, and low‐income areas. Among the articles with detailed genotype data, 98.04% (50/51) genotyped and/or sequenced the viral structural protein 1 (VP1) region of SaV. Overall, 14 distinct SaV genotypes were identified, including 7 GI genotypes, 6 GII genotypes, and 1 GIV genotype. Notably, GI.1 was the most prevalent genotype, followed by GI.2 and GII.1. Only one article reported genotyping and/or sequencing of both the VP1 and RNA‐dependent RNA polymerase (RdRp) regions, detecting genotypes GII.3[P3], GI.1[P1], GI.2[P2], GI.3[P3], GI.5[P5], GII.1[P1], GII.5[P5], GIV.1[P1], and GII.2[P3]. **Figure** **7** provides further details on the frequencies of SaV genogroups and genotypes reported in the included articles.

### 3.8 Sensitivity Analysis and Publication Bias

This study is a single‐group rate meta‐analysis, and its results are descriptive in nature rather than comparative. As single‐group rate meta‐analyses typically yield “positive” results, they are highly susceptible to publication bias. Notably, the pooled results demonstrated stability and statistical significance even when individual studies were removed from the analysis. In addition, sensitivity analysis and publication bias assessment using the “metaprop” command in STATA 12.0 software could not be executed due to the presence of boundary values (0% and 100%) in the dataset. Consequently, formal publication bias tests and sensitivity analyses were not performed.

## 4 Discussion

With the increasing utilization of more sensitive molecular techniques and the implementation of rotavirus vaccines, SaV has increasingly been recognized as a primary cause of sporadic and outbreak cases of AGE worldwide.^[^ ^7^, ^27^, ^28^, ^29^, ^30^ ^]^ Studies have demonstrated that SaV plays a significant role in AGE cases where NoV is not detected.^[^ ^12^ ^]^ Additionally, asymptomatic SaV carriers may contribute to secondary infections, leading to sporadic cases and outbreaks.^[^ ^31^ ^]^ Therefore, understanding the disease burden and molecular characteristics of SaV is crucial for developing effective prevention and management strategies. In this systematic review and meta‐analysis of 159 articles, we found that the prevalence of SaV in sporadic cases was 1.9%, while the asymptomatic prevalence in healthy control individuals was 0.8%. In SaV‐attributed outbreaks, the prevalence and asymptomatic prevalence were estimated at 16.4% and 14.4%, respectively. Furthermore, the detection rate of SaV was 58.8% in outbreak cases. The prevalence of SaV in sporadic cases (1.9%) was found to be lower than previous global estimates as well as those reported in Africa (3.4%, 2.2%, and 5%).^[^ ^1^, ^15^, ^32^ ^]^ Similarly, the asymptomatic prevalence of SaV (0.8%) in our study was lower than the 2.7% and 2% reported in previous studies.^[^ ^1^, ^32^ ^]^ Additionally, the prevalence of SaV in outbreak cases (16.4%) was lower than the 20.7% observed in prior research.^[^ ^27^ ^]^ This discrepancy may be attributed to China's rapid economic growth over the past three decades, which has enabled substantial improvements in drinking water quality and sanitation through sufficient funding and public health personnel allocation. Additionally, our findings indicate that the prevalence (1.9%) and asymptomatic prevalence (0.8%) of SaV in sporadic cases were significantly lower than those of NoV (11.5% and 7%, respectively).^[^ ^33^, ^34^ ^]^ Similarly, in outbreaks, the prevalence of SaV (16.4%) was lower than that of NoV (36.89%),^[^ ^35^ ^]^ and the asymptomatic prevalence of SaV (14.4%) was lower than that of NoV (17.6%).^[^ ^20^ ^]^ These observations suggest that SaV may have a comparatively weaker pathogenic mechanism, infectious dose, immune protection mechanism, and host susceptibility profile than NoV, despite both belonging to the human Caliciviridae family.

The prevalence of SaV in sporadic cases may be influenced by multiple factors. Subgroup analysis and meta‐regression were performed across eight collected variables. The findings indicate that the source of heterogeneity might be associated with the subject's age. The prevalence of SaV was found to begin rising from scattered children (2.9%), peak in kindergarten children (3.3%), subsequently fall to a trough in school‐age children (2%), and then experience a second peak in adults (2.2%). This pattern aligns with the epidemic characteristics of rotavirus and NoV, as all three viruses demonstrate higher prevalence in children under 5 years of age compared to adults.^[^ ^36^, ^37^ ^]^ These results provide evidence suggesting that childhood SaV infection may confer some protection against adult infection, thus indicating the potential for SaV vaccine development. Further analysis revealed similarities in the prevalence patterns of SaV and NoV. Both viruses showed a preschool‐age peak, a trough around the age of primary school students, and a second peak in adults and the elderly.^[^ ^38^ ^]^ This partially supports the hypothesis that SaV shares immune characteristics with norovirus, where immunity following infection can last longer than 8 years, rather than being short‐term (several months to 6 years).^[^ ^39^ ^]^ However, caution is needed when considering whether reinfection with a specific SaV genotype provides cross‐protection against other genotypes or genomes. Some studies have reported short‐term genotype‐specific immunity after SaV infection but no protection against other genotypes.^[^ ^27^, ^40^, ^41^ ^]^ It should be noted that superinfection with the same SaV genotype is rare, whereas superinfection involving the same genome is relatively more common.^[^ ^42^, ^43^ ^]^

In terms of geographical distribution, the prevalence of SaV in northern China (2.1%) and southern China (2%), as well as in inland regions (2.1%) and coastal areas (1.9%), showed remarkable comparability. Similarly, low‐income (2.2%), middle‐income (2.2%), and high‐income (1.9%) areas within China exhibited comparable SaV prevalence. These findings imply that further enhancing drinking water quality and sanitation measures alone may be insufficient to substantially reduce the potential for SaV transmission among the population.^[^ ^15^, ^44^ ^]^ Similar to NoV, SaV can be transmitted through contaminated food, particularly shellfish.^[^ ^45^ ^]^ Shellfish may become infected and serve as a reservoir for SaV, potentially leading to human exposure or consumption of contaminated shellfish, which can result in AGE.^[^ ^3^, ^45^ ^]^ Enhancing food safety could significantly alleviate the disease burden associated with SaV.^[^ ^32^ ^]^ High‐income regions, which have adequate public health funding and professionals to promote food safety, and most coastal provinces, which are high‐income areas, may partially explain the slightly lower SaV prevalence observed. The prevalence of SaV exhibits a seasonal pattern, with the highest occurrence during winter (2.8%), a temporal distribution closely resembling the epidemic characteristics of NoV‐induced AGE.^[^ ^3^, ^18^ ^]^ This seasonal peak may be attributed to increased indoor activities and close contact among individuals in crowded settings during winter, facilitating virus transmission via aerosol and direct contact routes. In China, the prevalence of SaV was higher among outpatients (2.2%) than inpatients (1.7%), a trend also reported by Diez Valcarce M. et al. (4% vs 2.3%) and Razizadeh MH. et al. (4% vs 2.4%).^[^ ^1^, ^15^ ^]^ A similar declining trend in NoV infection rates has been observed.^[^ ^36^ ^]^ This pattern may be explained by the fact that both SaV and NoV typically cause mild, self‐limiting illnesses that rarely require hospitalization. Regarding gender, the prevalence of SaV was higher in males (2.5%) than females (2.1%). Previous studies have identified male gender as a risk factor for acquiring AGE.^[^ ^46^, ^47^ ^]^ Potential explanations for this disparity include differences in sex hormone status (e.g., estrogen, testosterone), which play a crucial role in modulating host immunity against viral and bacterial infections,^[^ ^48^, ^49^, ^50^, ^51^, ^52^ ^]^ and the tendency for males to spend more time outdoors, increasing their exposure to pathogens.^[^ ^15^ ^]^ Close monitoring of SaV prevalence before and during the COVID‐19 pandemic (1.9% and 1.8%, respectively) suggests that non‐pharmaceutical interventions had a limited impact on the prevalence of SaV in sporadic cases.

This meta‐analysis revealed no significant association between SaV infection and sporadic AGE cases [OR: 1.30 (95% CI 0.83–2.05)]. However, the systematic review conducted by Razizadeh MH reported a significant correlation between SaV infection and sporadic AGE cases [OR: 1.84 (95% CI 1.27–2.66)].^[^ ^15^ ^]^ This discrepancy might stem from differences in the inclusion criteria of AGE cases across studies. Among the 135 routine surveillance articles in our meta‐analysis, 99 explicitly emphasized AGE cases with diarrhea but did not mention vomiting, while 36 articles specified AGE cases with diarrhea and/or vomiting. This suggests that numerous cases presenting solely with vomiting may have been excluded. Clinical manifestations vary among patients infected with different SaV genomes. Diarrhea tends to be more prevalent in patients infected with GI and GIV genomes, whereas vomiting is more common in those infected with GV genome strains.^[^ ^27^ ^]^

Genomic and genotypic identification are critical for analyzing currently circulating SaV strains within the human population.^[^ ^3^ ^]^ In this study, among sporadic cases with available SaV genome information, GI genomes (66.12%) were more prevalent than GII genomes (17.98%). The three most common genotypes identified in sporadic AGE cases were GI.1 (35.54%), GI.2 (24.59%), and GII.1 (11.98%). Similarly, across all included articles, GI genomes were reported more frequently (72.22%) compared to GII genomes (62.96%). This trend was consistent across high‐, middle‐, and low‐income areas. Among the 14 reported SaV genotypes, GI.1 was consistently the most prevalent, followed by GI.2 and GII.1. These findings indicate that both sporadic and outbreak cases of AGE are predominantly associated with the GI genome of SaV, as well as the GI.1, GI.2, and GII.1 genotypes. This aligns with long‐term surveillance results from Japan.^[^ ^29^ ^]^ Continuous surveillance of isolated viruses is essential for understanding SaV genotype distribution and provides valuable insights into transmission patterns, accurate diagnosis of circulating strains, population immunity assessment, and potential vaccine development opportunities.^[^ ^7^ ^]^ Based on our analysis, GI.1, GI.2, and GII.1 of SaV emerge as significant candidates for future vaccine development programs.

This systematic review, encompassing a comprehensive analysis of 159 studies across 32 Chinese provinces, provides a more precise estimation of SaV prevalence in sporadic and outbreak‐associated AGE cases. By synthesizing data from diverse sources and employing various study designs, the review enhances our understanding of SaV's epidemiological role. A key strength lies in its meticulous stratification analysis by age group, which is essential for discerning population susceptibility gradients and guiding epidemic prevention and control strategies. However, several limitations merit consideration. First, discrepancies in defining AGE cases across studies, particularly the underemphasis on vomiting as a symptom, likely led to underreporting of cases presenting solely with vomiting. Second, only 19 articles detailed their patient sampling methods, introducing potential methodological heterogeneity. Third, genotype information was provided in just 51 articles (32.08%), with 50 relying solely on VP1 region genes and only one utilizing both VP1 and RdRp region genes. Future research should systematically subtype both the VP1 and RdRp region genes to detect potential recombinant strains, as recombination events predominantly occur at the RdRp‐VP1 junction.^[^ ^53^ ^]^ Fourth, the geographical coverage of this review remains incomplete, with a notable research gap in middle‐income regions. Given the significant morbidity and mortality associated with AGE,^[^ ^1^, ^2^, ^54^ ^]^ expanding research efforts into these understudied areas will help provide more precise and representative national prevalence estimates of sapovirus infection. Fifth, the specific impact of SaV infection on the elderly remains unclear due to data conflation with adult populations. Given the high incidence of AGE in the elderly,^[^ ^55^ ^]^ dedicated studies on this age group are warranted. Sixth, the reporting of outcomes across multiple subgroups in many articles precluded multivariate meta‐regression analysis, though these findings still offer valuable preliminary insights for future comprehensive analyses of SaV transmission determinants. Seventh, substantial heterogeneity persists, potentially attributable to variations in laboratory protocols, sample sizes, AGE case definitions, and provincial public health policies, among other factors. While these factors could not be analyzed due to data limitations, they may have influenced prevalence estimates. Despite these limitations, this study offers critical insights into subgroup variations and overall prevalence patterns, including asymptomatic individuals, which are invaluable for disease burden estimation, vaccine development, and outbreak management strategies.

## 5 Conclusion

In sporadic cases, the overall prevalence of SaV was 1.9%, with an asymptomatic prevalence of 0.8%. However, during outbreaks, these figures rose significantly to 16.4% and 14.4%, respectively. SaV strains with GI genomes predominated in both sporadic and outbreak cases, with the genotypes GI.1, GI.2, and GII.1 being most prevalent. Notably, the prevalence of SaV showed an increasing trend from scattered children (2.9%), peaking among kindergarten children (3.3%). It then declined among school‐age children (2%) before reaching a second peak among adults (2.2%). These findings provide valuable insights for further investigations into the epidemiological characteristics and genotype distribution of human SaV infection. They also offer essential data for estimating disease burden, developing vaccines, formulating public health policies, and establishing emergency intervention strategies for SaV outbreaks.

## Conflict of Interest

The authors declare no conflict of interest.

## Author Contributions

T.‐T.Q. designed the study. Z.G. and X.‐J.Q. ran the search strategy, collected data, and re‐checked the data. T.‐J.L. performed statistical analyses. T.‐T.Q. checked the included articles and data. H.Z. checked the statistical analyses. X.‐Q.S. assessed the quality of studies, and S.‐S.P. confirmed the quality. Z.G. wrote the manuscript, and T.‐T.Q. edited the manuscript.

## Supporting information



Supporting Information

Supporting Information

## Data Availability

All data related to the study were included in the article or uploaded as Supporting Information. The datasets supporting the conclusions of this article are available through the published articles listed in Table 1, Table 2, and Additional File S1 (Supporting Information).

## References

[1] M. Diez Valcarce , A. K. Kambhampati , L. E. Calderwood , A. J. Hall , S. A. Mirza , J. Vinjé , PLoS One 2021, 16, 0255436. 10.1371/journal.pone.0255436 PMC8376006 34411109

[2] GBD 2019 Diseases and Injuries Collaborators , Lancet 2020, 396, 1204. 33069326 10.1016/S0140-6736(20)30925-9 PMC7567026

[3] T. Oka , Q. Wang , K. Katayama , L. J. Saif , Clin. Microbiol. Rev. 2015, 28, 32. 25567221 10.1128/CMR.00011-14 PMC4284302

[4] P. Mann , C. Pietsch , U. G. Liebert , Viruses 2019, 11, 726. 31394867 10.3390/v11080726 PMC6723979

[5] N. Lasure , V. Gopalkrishna , Epidemiol. Infect. 2017, 145, 106. 27609427 10.1017/S0950268816001953 PMC9507321

[6] J. R. Donowitz , J. Drew , M. Taniuchi , J. A. Platts‐Mills , M. Alam , T. Ferdous , T. Shama , M.d O. Islam , M. Kabir , U. Nayak , R. Haque , W. A. Petri , Clin. Infect. Dis. 2021, 73, 683. 10.1093/cid/ciaa1938 PMC8326554 33399861

[7] S. Becker‐Dreps , F. González , F. Bucardo , Curr. Opin. Infect. Dis. 2020, 33, 388. 32796163 10.1097/QCO.0000000000000671 PMC7748384

[8] Y. Jiao , T. Han , X. Qi , Y. Gao , J. Zhao , Y. Zhang , B. Li , Z. Zhang , J. Du , L. Sun , China CDC Wkly. 2023, 5, 625. 37520444 10.46234/ccdcw2023.119 PMC10372411

[9] P. Chirinda , F. Manjate , M. Garrine , M. A. Jr , N. Nobela , D. Vubil , T. Nhampossa , S. Acácio , Q. Bassat , K. L. Kotloff , M. M. Levine , J. P. Nataro , J. E. Tate , U. Parashar , J. M. Mwenda , P. L. Alonso , E. D. João , I. Mandomando , Viruses 2024, 16, 1159. 39066321 10.3390/v16071159 PMC11281453

[10] C. T. N. Mai , L. T. K. Ly , Y. H. Doan , T. Oka , L. T. P. Mai , N. T. Quyet , T. N. P. Mai , V. D. Thiem , L. T. Anh , L. Van Sanh , N. D. Hien , D. D. Anh , U. D. Parashar , J. E. Tate , N. Van Trang , Viruses 2023, 15, 2164. 38005842 10.3390/v15112164 PMC10675811

[11] J. A. Platts‐Mills , J. Liu , E. T. Rogawski , F. Kabir , P. Lertsethtakarn , M. Siguas , S. S. Khan , I. Praharaj , A. Murei , R. Nshama , B. Mujaga , A. Havt , I. A. Maciel , T. L. McMurry , D. J. Operario , M. Taniuchi , J. Gratz , S. E. Stroup , J. H. Roberts , A. Kalam , F. Aziz , S. Qureshi , M. O. Islam , P. Sakpaisal , S. Silapong , P. P. Yori , R. Rajendiran , B. Benny , M. McGrath , B. J. J. McCormick , et al., Lancet Global Health 2018, 6, 1309.

[12] M. Magwalivha , J. P. Kabue , A. N. Traore , N. Potgieter , Adv. Virol. 2018, 2018, 5986549. 30245718 10.1155/2018/5986549 PMC6139206

[13] S. M. Ahmed , B. A. Lopman , K. Levy , PLoS One 2013, 8, 75922. 10.1371/journal.pone.0075922 PMC3788804 24098406

[14] J. Liu , J. Gratz , C. Amour , G. Kibiki , S. Becker , L. Janaki , J. J. Verweij , M. Taniuchi , S. U. Sobuz , R. Haque , D. M. Haverstick , E. R. Houpt , J. Clin. Microbiol. 2013, 51, 472. 23175269 10.1128/JCM.02658-12 PMC3553916

[15] M. H. Razizadeh , A. Khatami , M. Zarei , Rev. Med. Virol. 2022, 32, 2302. 10.1002/rmv.2302 34626019

[16] C. A. Omatola , R. E. Ogunsakin , A. B. Onoja , M.‐L. O. Okolo , J. Abraham‐Oyiguh , K. C. Mofolorunsho , P. Q. Akoh , O. P. Adejo , J. Idakwo , T. O. Okeme , D. Muhammed , D. M. Adaji , S. O. Samson , R. F. Aminu , M. E. Akor , E. Edegbo , A. M. Adamu , J. Infect. 2024, 88, 106169. 38697269 10.1016/j.jinf.2024.106169

[17] M. J. Page , J. E. McKenzie , P. M. Bossuyt , I. Boutron , T. C. Hoffmann , C. D. Mulrow , L. Shamseer , J. M. Tetzlaff , E. A. Akl , S. E. Brennan , R. Chou , J. Glanville , J. M. Grimshaw , A. Hróbjartsson , M. M. Lalu , T. Li , E. W. Loder , E. Mayo‐Wilson , S. McDonald , L. A. McGuinness , L. A. Stewart , J. Thomas , A. C. Tricco , V. A. Welch , P. Whiting , D. Moher , BMJ 2021, 372, n71. 33782057 10.1136/bmj.n71 PMC8005924

[18] Chinese Center for Disease Control and Prevention , Chin. J. Prev. Med. 2016, 1, 7. 10.46234/ccdcw2020.084 PMC8392945 34594650

[19] National Bureau of Statistics of China , Statistical system and classification standard, http://www.stats.gov.cn/zs/tjws/cjwtjd/202302/t20230217_1912798.html (accessed: August 2022).

[20] J. Wang , Z. H. Ji , S. B. Zhang , Z. R. Yang , X. Q. Sun , H. Zhang , J. Med. Virol. 2024, 96, 29393. 10.1002/jmv.29393 38235934

[21] Z. Munn , S. Moola , K. Lisy , D. Riitano , C. Tufanaru , in JBI Manual for Evidence Synthesis (Eds: T. Aromataris , Z. Munn ), Joanna Briggs Institute, Adelaide, Australia 2020.

[22] P. Ho , M. Bulsara , J. Downs , S. Patman , C. Bulsara , A. M. Hill , JBI Database Syst. Rev. Implement. Rep. 2019, 17, 390. 10.11124/JBISRIR-2017-003798 30870331

[23] Z. Ding , Y. Zhai , C. Wu , H. Wu , Q. Lu , J. Lin , F. He , J. Epidemiol. 2017, 27, 274. 28457602 10.1016/j.je.2016.07.006 PMC5463023

[24] J. P. Higgins , S. G. Thompson , J. J. Deeks , D. G. Altman , BMJ 2003, 327, 557. 12958120 10.1136/bmj.327.7414.557 PMC192859

[25] J. Morze , C. Wittenbecher , L. Schwingshackl , A. Danielewicz , A. Rynkiewicz , F. B. Hu , M. Guasch‐Ferré , Diabetes Care 2022, 45, 1013. 35349649 10.2337/dc21-1705 PMC9016744

[26] M. Farahmand , M. Moghoofei , A. Dorost , Z. Shoja , S. Ghorbani , S. J. Kiani , P. Khales , A. Esteghamati , S. Sayyahfar , M. Jafarzadeh , S. Minaeian , K. Khanaliha , M. Naghdalipour , A. Tavakoli , Rev. Med. Virol. 2022, 32, 2237. 10.1002/rmv.2237 33793023

[27] Y. Yu , X. H. Guo , H. Q. Yan , Z. Y. Gao , W. H. Li , B. W. Liu , Q. Y. Wang , Chin. J. Epidemiol. 2019, 40, 93. 10.3760/cma.j.issn.0254-6450.2019.01.019 30669739

[28] S. Becker‐Dreps , F. Bucardo , S. Vilchez , L. E. Zambrana , L. Liu , D. J. Weber , R. Peña , L. Barclay , J. Vinjé , M. G. Hudgens , J. Nordgren , L. Svensson , D. R. Morgan , F. Espinoza , M. Paniagua , Pediatr. Infect. Dis. J. 2014, 33, 1156. 24879131 10.1097/INF.0000000000000427 PMC4216626

[29] S. A. Hoque , K. Nishimura , A. Thongprachum , P. Khamrin , N. Thi Kim Pham , M. T. Islam , N. Khandoker , S. Okitsu , Y. Onda‐Shimizu , S. K. Dey , N. Maneekarn , T. Kobayashi , S. Hayakawa , H. Ushijima , J. Infect. Public Health 2022, 15, 315. 35124328 10.1016/j.jiph.2022.01.019

[30] F. Bucardo , Y. Reyes , L. Svensson , J. Nordgren , PLoS One 2014, 9, 98201. 10.1371/journal.pone.0098201 PMC4029982 24849288

[31] F. González , M. Diez‐Valcarce , Y. Reyes , N. A. Vielot , C. Toval‐Ruíz , L. Gutiérrez , O. Zepeda , E. C. Cuadra , P. Blandón , H. Browne , N. M. Bowman , S. Vílchez , J. Vinjé , S. Becker‐Dreps , F. Bucardo , Clin. Microbiol. Infect. 2023, 29, 540.e9. 10.1016/j.cmi.2022.11.013 PMC10077563 36423864

[32] K. Makhaola , S. Moyo , L. P. Kebaabetswe , Viruses 2020, 12, 490. 32349380 10.3390/v12050490 PMC7291139

[33] T.‐T. Li , Q. Xu , M.‐C. Liu , T. Wang , T.‐L. Che , A.‐Y. Teng , C.‐L. Lv , G.‐L. Wang , F. Hong , W. Liu , L.i‐Q. Fang , Viruses 2023, 15, 1336. 37376635 10.3390/v15061336 PMC10302178

[34] R. Qi , Y. T. Huang , J. W. Liu , Y. Sun , X. F. Sun , H. J. Han , X. R. Qin , M. Zhao , L. J. Wang , W. Li , J. H. Li , C. Chen , X. J. Yu , EClinicalMedicine 2018, 2‐3, 50. 10.1016/j.eclinm.2018.09.001 PMC6537540 31193628

[35] P. Zhang , C. Hao , X. Di , X. Chuizhao , L. Jinsong , Z. Guisen , L. Hui , D. Zhaojun , Front Public Health 2024, 12, 1373322. 38993708 10.3389/fpubh.2024.1373322 PMC11236571

[36] Y. Liao , X. Hong , A. Wu , Y. Jiang , Y. Liang , J. Gao , L. Xue , X. Kou , Microb. Pathog. 2021, 161, 105259 34687838 10.1016/j.micpath.2021.105259

[37] C. Thystrup , S. E. Majowicz , D. B. Kitila , B. N. Desta , O. E. Fayemi , C. I. Ayolabi , E. Hugho , E. M. Buys , G. B. Akanni , N. E. Machava , C. Monjane , T. Hald , S. M. Pires , BMC Public Health 2024, 24, 1864. 38997671 10.1186/s12889-024-19334-8 PMC11241906

[38] H.‐L. Zhou , S.‐S. Zhen , J.‐X. Wang , C.‐J. Zhang , C. Qiu , S.‐M. Wang , X. Jiang , X.‐Y. Wang , J. Infect. 2017, 75, 216. 28633888 10.1016/j.jinf.2017.06.004

[39] K. Simmons , M. Gambhir , J. Leon , B. Lopman , Emerging Infect. Dis. 2013, 19, 1260. 10.3201/eid1908.130472 PMC3739512 23876612

[40] S. Harada , T. Oka , E. Tokuoka , N. Kiyota , K. Nishimura , Y. Shimada , T. Ueno , S. Ikezawa , T. Wakita , Q. Wang , L. J. Saif , K. Katayama , Arch. Virol. 2012, 157, 1999. 22772483 10.1007/s00705-012-1387-7

[41] G. J. Sánchez , H. Mayta , M. J. Pajuelo , K. Neira , L. Xiaofang , L. Cabrera , S. B. Ballard , J. E. Crabtree , D. Kelleher , V. Cama , C. Bern , H. Oshitani , R. H. Gilman , M. Saito , M. Ochoa , M. Vittet , A. Pando , Clin. Infect. Dis. 2018, 66, 1858. 29309577 10.1093/cid/cix1103 PMC5982808

[42] V. K. Menon , S. George , R. Sarkar , S. Giri , P. Samuel , R. Vivek , A. Saravanabavan , F. B. Liakath , S. Ramani , M. Iturriza‐Gomara , J. J. Gray , D. W. Brown , M. K. Estes , G. Kang , PLoS One 2016, 11, 0157007. 10.1371/journal.pone.0157007 PMC4902233 27284939

[43] M. Saito , S. Goel‐Apaza , S. Espetia , D. Velasquez , L. Cabrera , S. Loli , J. E. Crabtree , R. E. Black , M. Kosek , W. Checkley , M. Zimic , C. Bern , V. Cama , R. H. Gilman , L. Xiao , D. Kelleher , H. J. Windle , L. J. van Doorn , M. Varela , M. Verastegui , M. Calderon , A. Alva , K. Roman , Clin. Infect. Dis. 2014, 58, 483. 24300042 10.1093/cid/cit763 PMC3905757

[44] S. Becker‐Dreps , F. Bucardo , J. Vinjé , Lancet Child Adolesc. Health 2019, 3, 758. 31439497 10.1016/S2352-4642(19)30270-6 PMC7213765

[45] C. P. Cantelli , G. C. L. Tavares , S. K. Sarmento , F. M. Burlandy , T. M. Fumian , A. G. Maranhão , E. S. R. F. D. Silva , M. A. P. Horta , M. P. Miagostovich , Z. Yang , J. P. G. Leit , Viruses 2024, 16, 317.38543684

[46] S. de Lusignan , J. Sherlock , F. Ferreira , S. O'Brien , M. Joy , BMC Public Health 2020, 20, 445. 32248812 10.1186/s12889-020-08525-8 PMC7132989

[47] C.‐J. Chen , F.‐T. Wu , Y.‐C. Huang , W.‐C. Chang , H.‐S. Wu , C.‐Y. Wu , J.‐S. Lin , F.‐C. Huang , C. A. Hsiung , Medicine 2015, 94, 1372.

[48] L. M. Pennell , C. L. Galligan , E. N. Fish , J. Autoimmun. 2012, 38, J282. 22225601 10.1016/j.jaut.2011.11.013

[49] S. P. Dias , M. C. Brouwer , Infect. Immun. 2022, 90, 0028322. 10.1128/iai.00283-22 PMC9584217 36121220

[50] R. H. Straub , Endocr. Rev. 2007, 28, 521.. 17640948 10.1210/er.2007-0001

[51] B. A. Araneo , T. Dowell , M. Diegel , R. A. Daynes , Blood 1991, 78, 688. 1830499

[52] M. V. Scalerandi , N. Peinetti , C. Leimgruber , M. M. Cuello Rubio , J. P. Nicola , G. B. Menezes , C. A. Maldonado , A. A. Quintar , Front. Immunol. 2018, 9, 1980. 30233581 10.3389/fimmu.2018.01980 PMC6129603

[53] K. Dos Anjos , L. M. Lima , P. A. Silva , Arch. Virol. 2011, 156, 1953. 21796399 10.1007/s00705-011-1079-8

[54] World Health Organization . Diarrhoeal disease, https://www.who.int/news‐room/fact‐sheets/detail/diarrhoeal‐disease (accepted: March 2024).

[55] M. Krutova , A. Briksi , J. Tkadlec , M. Zajac , J. Matejkova , O. Nyc , P. Drevinek , J. Clin. Microbiol. 2019, 57, 00710. 10.1128/JCM.00710-19 PMC6760961 31391230

