# Supplementary material for: Prevalence and Genotype Distribution of Sapovirus in China: A Systematic Review and Meta‐Analysis
Source: Glob Chall. 2025 Aug 5;9(9):e00369. doi: 10.1002/gch2.202400369 (PMC12418334; doi:10.1002/gch2.202400369)
Supplement: Supplementary file 1 — Supporting Information [file GCH2-9-e00369-s002.doc]

**Supplementary Material**

**Prevalence and genotype distribution of sapovirus in China: A systematic review and meta-analysis**

**Contents**

[Supplementary Table S1: Preferred Reporting Items for Systematic reviews and Meta-Analyses (PRISMA) 2020 Checklist 2](#__RefHeading___Toc130148038)

[Supplementary Table S2: Search strategy for PubMed database 6](#__RefHeading___Toc130148038)

[Supplementary Table S3: Search strategy for Web of Science database 6](#__RefHeading___Toc130148038)

[Supplementary Figure S1: Asymptomatic prevalence of SaV in non-AGE control individuals 7](#__RefHeading___Toc130148040)

[Supplementary Figure S2: Detection rate of SaV among cases in outbreaks.](#__RefHeading___Toc130148040) 8

[Supplementary Figure S3: Asymptomatic prevalence of SaV among exposed individuals in outbreaks](#__RefHeading___Toc130148040) 9

[Supplementary Figure S4: Sensitivity analysis of meta-analysis by R software on routine surveillance articles 1](#__RefHeading___Toc130148040)0

[Supplementary Figure S5: Funnel plot for assessment of publication bias by R software on routine surveillance articles 1](#__RefHeading___Toc130148040)1

[Supplementary Figure S6: Sensitivity analysis of meta-analysis by R software on outbreak articles 1](#__RefHeading___Toc130148040)2

[Supplementary Figure S7: Funnel plot for assessment of publication bias by R software on outbreak articles 1](#__RefHeading___Toc130148040)3

[Included Articles 1](#__RefHeading___Toc130148046)4

**Supplementary Table S1:** Preferred Reporting Items for Systematic reviews and Meta-Analyses (PRISMA) 2020 Checklist

| **Section and Topic** | **Item #** | **Checklist item** | **Location where item is reported** |
| --- | --- | --- | --- |
| **TITLE** | | |  |
| Title | 1 | Identify the report as a systematic review. | 1 |
| **ABSTRACT** | | |  |
| Abstract | 2 | See the PRISMA 2020 for Abstracts checklist. | 2 |
| **INTRODUCTION** | | |  |
| Rationale | 3 | Describe the rationale for the review in the context of existing knowledge. | 3-4 |
| Objectives | 4 | Provide an explicit statement of the objective(s) or question(s) the review addresses. | 5-6 |
| **METHODS** | | |  |
| Eligibility criteria | 5 | Specify the inclusion and exclusion criteria for the review and how studies were grouped for the syntheses. | 7-8 |
| Information sources | 6 | Specify all databases, registers, websites, organisations, reference lists and other sources searched or consulted to identify studies. Specify the date when each source was last searched or consulted. | 6 |
| Search strategy | 7 | Present the full search strategies for all databases, registers and websites, including any filters and limits used. | 6-7;Supplementary Table S2,S3 |
| Selection process | 8 | Specify the methods used to decide whether a study met the inclusion criteria of the review, including how many reviewers screened each record and each report retrieved, whether they worked independently, and if applicable, details of automation tools used in the process. | 7-8 |
| Data collection process | 9 | Specify the methods used to collect data from reports, including how many reviewers collected data from each report, whether they worked independently, any processes for obtaining or confirming data from study investigators, and if applicable, details of automation tools used in the process. | 8-9 |
| Data items | 10a | List and define all outcomes for which data were sought. Specify whether all results that were compatible with each outcome domain in each study were sought (e.g. for all measures, time points, analyses), and if not, the methods used to decide which results to collect. | 8 |
|  | 10b | List and define all other variables for which data were sought (e.g. participant and intervention characteristics, funding sources). Describe any assumptions made about any missing or unclear information. | 7-10 |
| Study risk of bias assessment | 11 | Specify the methods used to assess risk of bias in the included studies, including details of the tool(s) used, how many reviewers assessed each study and whether they worked independently, and if applicable, details of automation tools used in the process. | 18;Supplementary Figure S5,S7 |
| Effect measures | 12 | Specify for each outcome the effect measure(s) (e.g. risk ratio, mean difference) used in the synthesis or presentation of results. | 8 |
| Synthesis methods | 13a | Describe the processes used to decide which studies were eligible for each synthesis (e.g. tabulating the study intervention characteristics and comparing against the planned groups for each synthesis (item #5)). | 8-10 |
|  | 13b | Describe any methods required to prepare the data for presentation or synthesis, such as handling of missing summary statistics, or data conversions. | 10-11 |
|  | 13c | Describe any methods used to tabulate or visually display results of individual studies and syntheses. | 10-11 |
|  | 13d | Describe any methods used to synthesize results and provide a rationale for the choice(s). If meta-analysis was performed, describe the model(s), method(s) to identify the presence and extent of statistical heterogeneity, and software package(s) used. | 10-11 |
|  | 13e | Describe any methods used to explore possible causes of heterogeneity among study results (e.g. subgroup analysis, meta-regression). | 10-11 |
|  | 13f | Describe any sensitivity analyses conducted to assess robustness of the synthesized results. | 18;Supplementary Figure S4,S6 |
| Reporting bias assessment | 14 | Describe any methods used to assess risk of bias due to missing results in a synthesis (arising from reporting biases). | 10-11;Supplementary Figure S5,S7 |
| Certainty assessment | 15 | Describe any methods used to assess certainty (or confidence) in the body of evidence for an outcome. | N/A |
| **RESULTS** | | |  |
| Study selection | 16a | Describe the results of the search and selection process, from the number of records identified in the search to the number of studies included in the review, ideally using a flow diagram. | 11-12; Figure 1 |
|  | 16b | Cite studies that might appear to meet the inclusion criteria, but which were excluded, and explain why they were excluded. | 11-12 |
| Study characteristics | 17 | Cite each included study and present its characteristics. | 12-14; Table 1 and 2; Figure 2 |
| Risk of bias in studies | 18 | Present assessments of risk of bias for each included study. | 18;Supplementary Figure S5,S7 |
| Results of individual studies | 19 | For all outcomes, present, for each study: (a) summary statistics for each group (where appropriate) and (b) an effect estimate and its precision (e.g. confidence/credible interval), ideally using structured tables or plots. | 14-17; Figure 3,4,5,6;Supplementary Figure S1,S2,S3 |
| Results of syntheses | 20a | For each synthesis, briefly summarise the characteristics and risk of bias among contributing studies. | 14-17;Supplementary Figure S5,S7 |
|  | 20b | Present results of all statistical syntheses conducted. If meta-analysis was done, present for each the summary estimate and its precision (e.g. confidence/credible interval) and measures of statistical heterogeneity. If comparing groups, describe the direction of the effect. | 14-17; Figure 3,4,5,6;Supplementary Figure S1,S2,S3 |
|  | 20c | Present results of all investigations of possible causes of heterogeneity among study results. | 15-16 |
|  | 20d | Present results of all sensitivity analyses conducted to assess the robustness of the synthesized results. | 18;Supplementary Figure S4,S6 |
| Reporting biases | 21 | Present assessments of risk of bias due to missing results (arising from reporting biases) for each synthesis assessed. | 18;Supplementary Figure S5,S7 |
| Certainty of evidence | 22 | Present assessments of certainty (or confidence) in the body of evidence for each outcome assessed. | 14-17 |
| **DISCUSSION** | | |  |
| Discussion | 23a | Provide a general interpretation of the results in the context of other evidence. | 19-25 |
|  | 23b | Discuss any limitations of the evidence included in the review. | 25-27 |
|  | 23c | Discuss any limitations of the review processes used. | 25-27 |
|  | 23d | Discuss implications of the results for practice, policy, and future research. | 27 |
| **OTHER INFORMATION** | | |  |
| Registration and protocol | 24a | Provide registration information for the review, including register name and registration number, or state that the review was not registered. | 6 |
|  | 24b | Indicate where the review protocol can be accessed, or state that a protocol was not prepared. | N/A |
|  | 24c | Describe and explain any amendments to information provided at registration or in the protocol. | N/A |
| Support | 25 | Describe sources of financial or non-financial support for the review, and the role of the funders or sponsors in the review. | 28 |
| Competing interests | 26 | Declare any competing interests of review authors. | 28 |
| Availability of data, code and other materials | 27 | Report which of the following are publicly available and where they can be found: template data collection forms; data extracted from included studies; data used for all analyses; analytic code; any other materials used in the review. | 28 |

*From:*  Page MJ, McKenzie JE, Bossuyt PM, Boutron I, Hoffmann TC, Mulrow CD, et al. The PRISMA 2020 statement: an updated guideline for reporting systematic reviews. BMJ 2021;372:n71. doi: 10.1136/bmj.n71

**Supplementary Table S2:** Search strategy for PubMed

| # | Searches | Results |
| --- | --- | --- |
| 1 | "Sapovirus"[MeSH Terms] | 439 |
| 2 | "sapovirus*"[Title/Abstract] | 849 |
| 3 | "Sapovirus"[MeSH Terms] OR "sapovirus*"[Title/Abstract] | 880 |
| 4 | "china"[Title/Abstract] OR "hong kong"[Title/Abstract] OR "Taiwan"[Title/Abstract] OR "Macau"[Title/Abstract] | 396,333 |
| 5 | ("china"[Title/Abstract] OR "hong kong"[Title/Abstract] OR "Taiwan"[Title/Abstract] OR "Macau"[Title/Abstract]) AND ("Sapovirus"[MeSH Terms] OR "sapovirus*"[Title/Abstract]) | 84 |

**Supplementary Table S3:** Search strategy for Web of Science

| # | Searches | Results |
| --- | --- | --- |
| 1 | ALL=(sapovirus*) | 819 |
| 2 | ALL=(infect*) | 1,920,601 |
| 3 | (((ALL=(china)) OR ALL=(Hong Kong)) OR ALL=(Taiwan)) OR ALL=(Macau) | 6,950,427 |
| 4 | #1 AND #2 AND #3 | 113 |


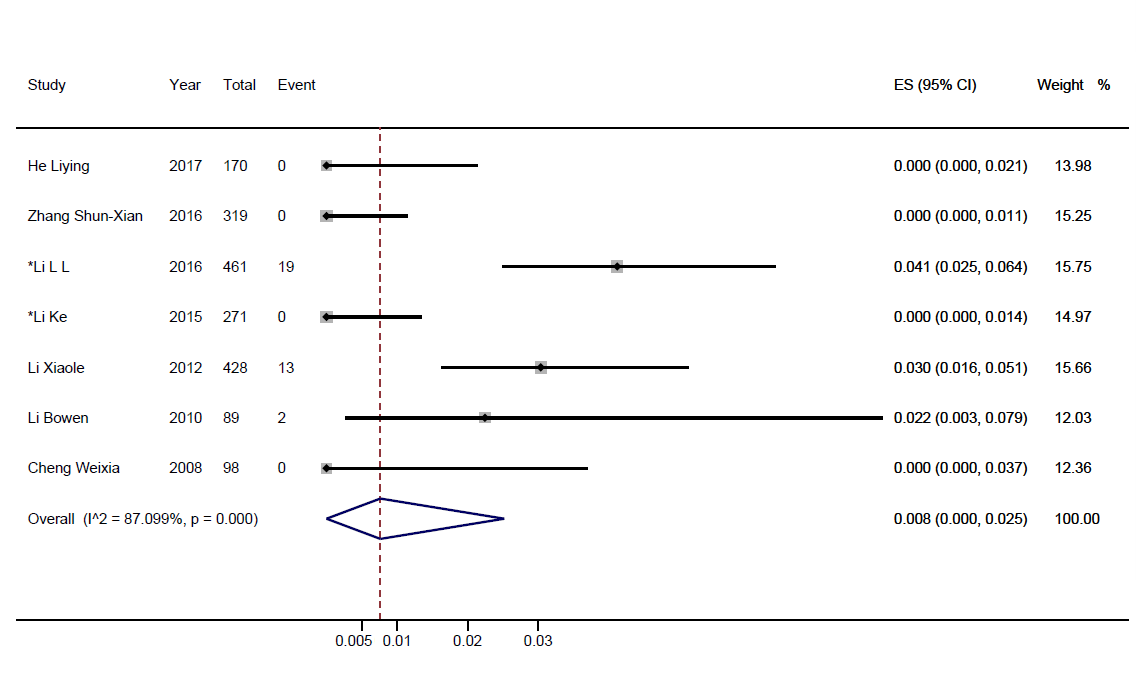
**Supplementary Figure S1.** Forest graph: Asymptomatic prevalence of SaV in non-AGE control individuals in seven routine surveillance articles. Event: Number of SaV-positive asymptomatic individuals. Total: Number of asymptomatic individuals whose samples were detected.


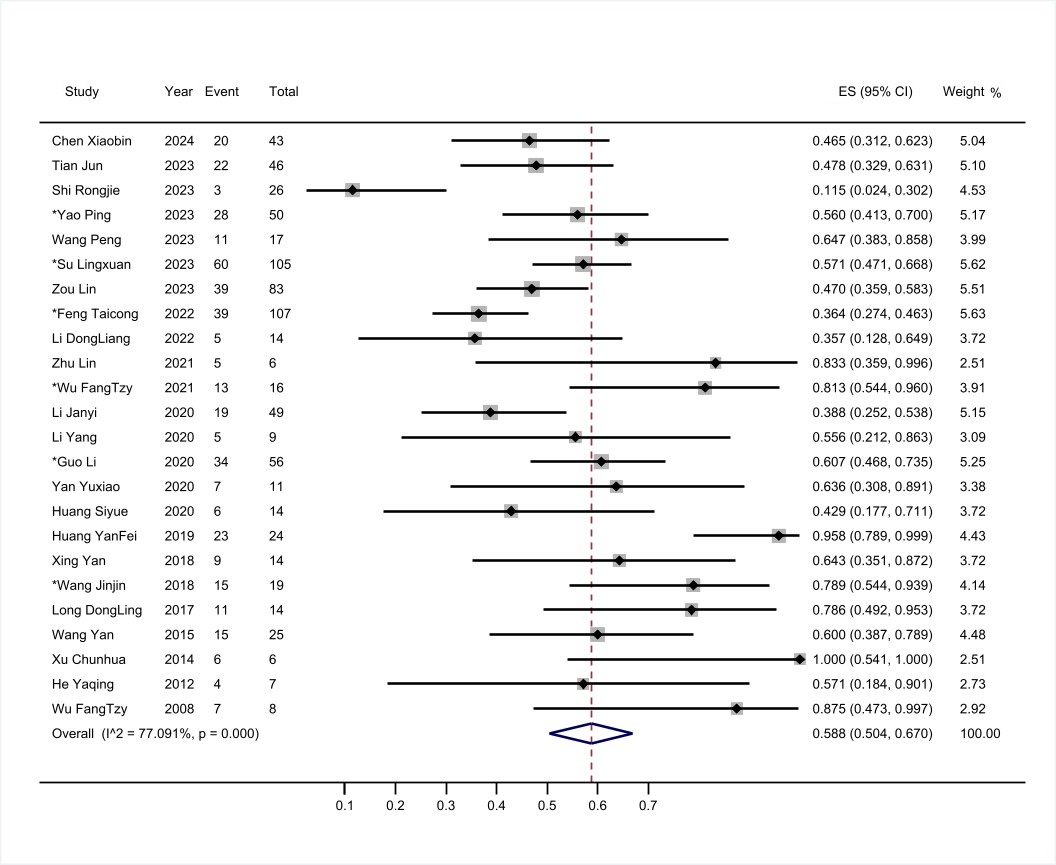
**Supplementary Figure S2.** Forest graph: the pooled detection rate of SaV among AGE cases in 24 outbreak articles. Event: Number of SaV-positive cases. Total: Number of cases whose samples were tested.

**Supplementary Figure S3.** Forest graph: Asymptomatic prevalence of SaV among exposed individuals in 11 outbreak articles. Event: Number of SaV-positive asymptomatic individuals. Total: Number of exposed asymptomatic individuals whose samples were detected.

**
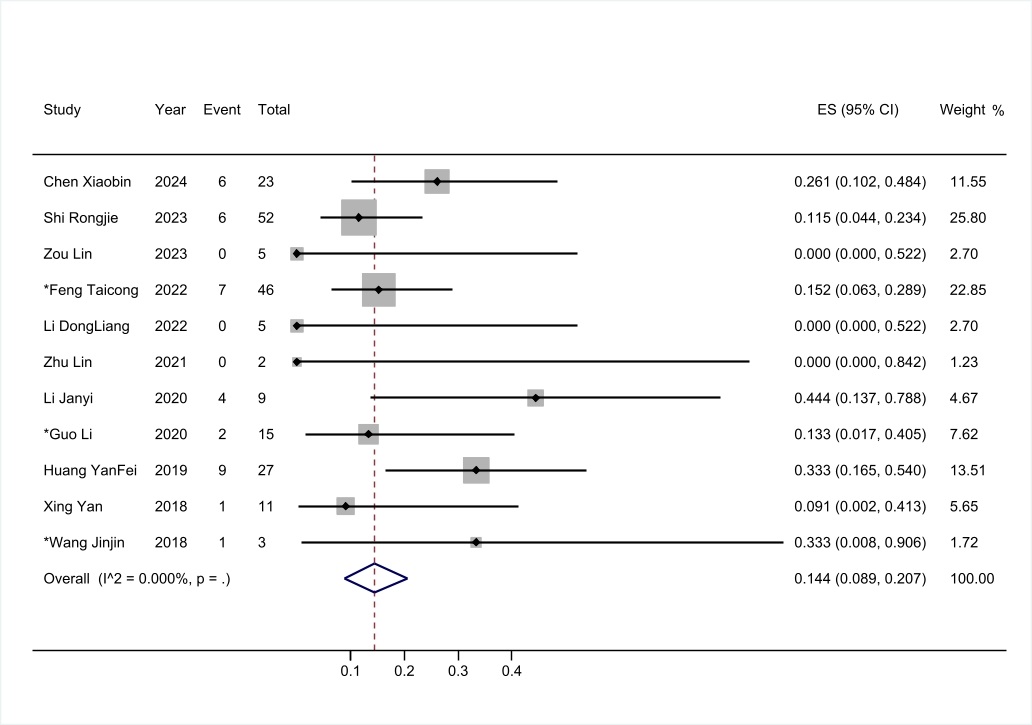
**

**Supplementary Figure S4.** Sensitivity analysis of meta-analysis in 135 routine surveillance articles (carried out by omitting one study at a time) (By R software)

**
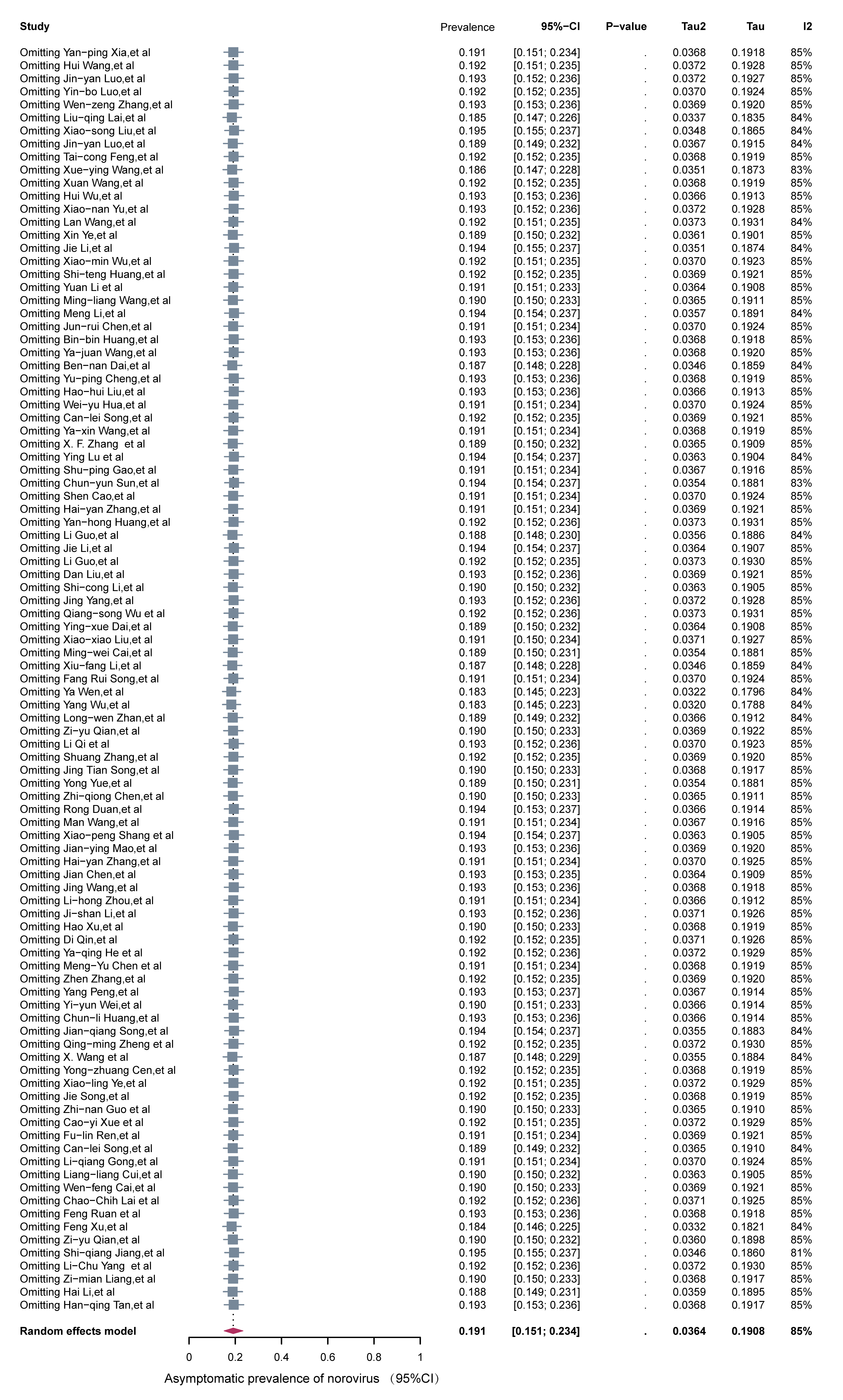
**

**Supplementary Figure S5.** Funnel plot for assessment of publication bias in 135 routine surveillance articles (Peter's test, *p* =0.005) (By R software)

**
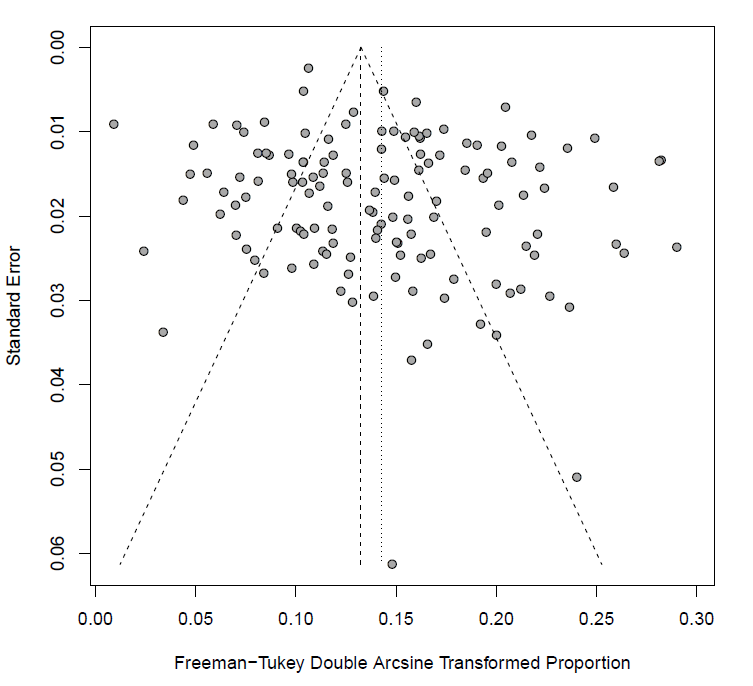
**

**Supplementary Figure S6.** Sensitivity analysis of meta-analysis in 24 outbreak articles (carried out by omitting one study at a time) (By R software)

**
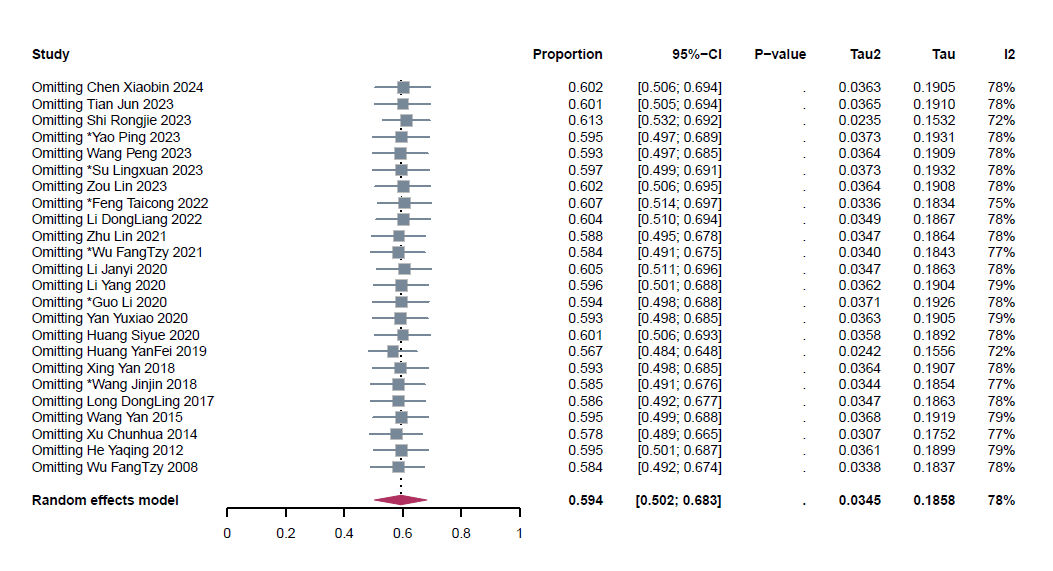
**

**Supplementary Figure S7.** Funnel plot for assessment of publication bias in 24 outbreak articles (Peter's test, *p* = 0.197) (By R software)

**
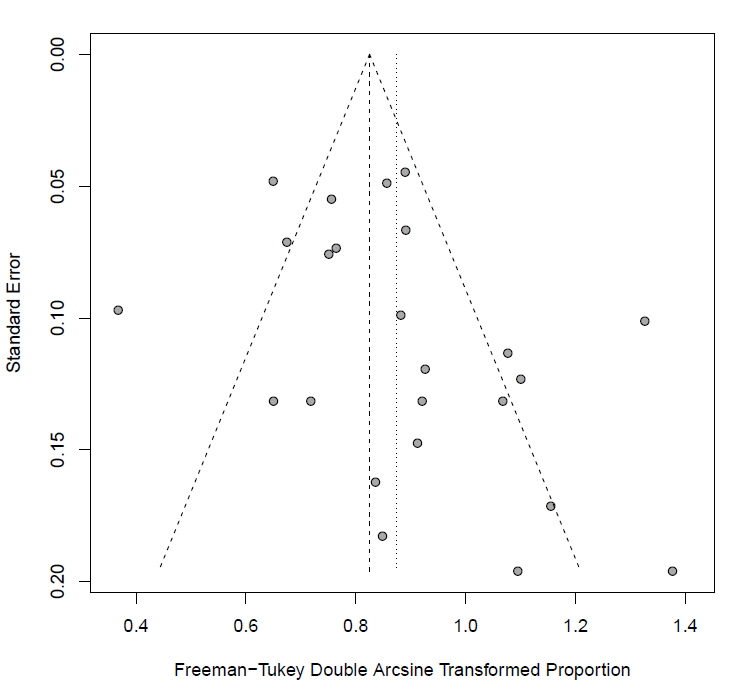
**

**Included Articles**

**Routine surveillance studies**

[1] Kuang XZ, Xiao WJ, Pan H,et al. Etiological characteristics of viral gastroenteritis in pediatric inpatients under five years old in Shanghai, 2021‒2022[J]. Shanghai J Prev Med,2024,36(02):143-149.doi:10.19428/j.cnki.sjpm.2024.23217. In Chinese.

[2] Wu Q, Zhang YG, Yun RT. Monitoring of prevalence of diarrhea among children aged less than 5 years old in a sentinel monitoring hospital of Hohhot from 2020 to 2022[J/OL].Chin J Nosocomiol,2024(10):1558-1562. In Chinese.

[3] Wu JM, Zhang YG, Song J,et al. Pathogen surveillance analysis of hospitalized infants with viral diarrhea in Hohhot from 2016 to 2020[J].J Med Pest Control,2024,40(04):372-375. In Chinese.

[4] Xu K, Feng SQ, Su LY,et al. Pathogenic Surveillance on the Viral Diarrhea in Chengdu from 2021 to 2022[J].J Prev Med Inf,2024,40(04):409-417.doi:10.19971/j.cnki.1006-4028.230221. In Chinese.

[5] Ji X, Guo C, Dai Y, et al. Genomic Characterization and Molecular Evolution of Sapovirus in Children under 5 Years of Age. Viruses. 2024;16(1):146. doi: 10.3390/v16010146.

[6] Dong M, Liu HB, Wang YX,et al.Analysis of intestinal pathogen infection in diarrhea cases under the regular prevention and control of COVID-19 in Fangshan District[J].J Med Pest Control,2023,39(12):1137-1140. In Chinese.

[7] Geng XY. Epidemiological characteristics of adult infectious diarrhea in an area of Shanghai and the construction of a predictive model[D].Nanchang University,2024.Dissertation. doi:10.27232/d.cnki.gnchu.2023.000758. In Chinese.

[8] Huang XL. Molecular epidemiology of them norovirus based on the sentinel Hospitals in three cities located in Shandong Province[D]. Shandong University,2024.Dissertation. doi:10.27272/d.cnki.gshdu.2023.001713. In Chinese.

[9] Jiao Y, Guo L, Han TL,et al. Analysis of the characteristics of viral infections in children with diarrhea in Beijing from 2018 to 2022[J].Chinese J Prev Med,2023,57(7):976-982. doi:10.3760/cma.j.cn112150-20230131-00066. In Chinese.

[10] Kang Q, Lv CX, Wang XY,et al. Epidemiological characteristics of viruses causing diarrhea and whole-genome features of rotavirus A in Gansu,2018−2020[J]. Dis surveill, 2023,38(02):174-180. In Chinese.

[11] Li S, Gao ZY, Li WH, et al. Gene characteristics analysis on Sapovirus from diarrhea cases in Beijing, 2019[J]. Chin J Epidemiol, 2023,44(11):1787-1794. doi:10.3760/cma.j.cn112338-20230505-00278. In Chinese.

[12] Liang YF. Pathogen spectrum analysis with infectious diarrhea in children and identiflcation of Sapovirus in Guangzhou,2021-2022[D].Southern Medical University,2024.Dissertation. doi:10.27003/d.cnki.gojyu.2023.000276. In Chinese.

[13] Liu MQ, Chen JK, Huang ZM, et al. Surveillance results of viral diarrhea in children under five years of age in Shaoxing City[J]. Prev Med,2023,35(10):903-906. doi:10.19485/j.cnki.issn2096-5087.2023.10.016. In Chinese.

[14] Luo SH, Feng B, Yao W, et al. Etiological and epidemiological characteristics of viral infectious diarrhea in Guangming district of Shenzhen,Guangdong, 2018−2021[J].Dis surveill, 2023,38(06):694-700. In Chinese.

[15] Shabiremu Tohetamu, Wang T, Mistral Nigati,et al. Etiological characteristics of viral diarrhea in hospitalized children under 5 years old in Urumqi, Xinjiang, 2018−2021[J].Dis surveill, 2023,38(07):842-847. In Chinese.

[16] Wu BS, Huang MZ, Lin WD, et al. Molecular prevalent characteristics of GII norovirus in children under 5 years of age in Fuzhou from 2016 to 2021[J].Chin J Zoonoses, 2023,39(05):471-477. In Chinese.

[17] Yao W, Du HR, Li Y, et al. Surveillance and analysis of infectious diarrhea pathogens in sentinel surveillance hospitals in Guangming district，Shenzhen from 2015 to 2021[J].J Trop Med, 2023,23(10):1477-1480. In Chinese.

[18] Zhang Q, Deng F. Etiological surveillance of children with diarrhea hospitalized in Anhui Children’s Hospital from 2016 to 2021[J].Chin Frontier Health Quarantine, 2023,46(01):74-78. doi:10.16408/j.1004-9770.2023.01.017. In Chinese.

[19] Gao Q, Liu H, Yu W, et al. Pathogenetic characteristics of infectious diarrhea in Yantai City, Shandong Province, 2018-2019. Front Public Health. 2023;11:1195118. doi: 10.3389/fpubh.2023.1195118.

[20] Chen CP, Xie P, Bai R, et al. Analysis on clinical characteristics in 464 patients over 14 years old with infectious diarrhea[J]. Lab Med Cli,2022,19(4):433-437,442. doi:10.3969/j.issn.1672-9455.2022.04.001. In Chinese.

[21] Chen S, Liu Q, Xu YJ, et al. Analysis on surveillance results of viral diarrhea among children under 14 years old in sentinel hospital of Fengxian District in Shanghai from 2016-2019[J].Occup and Health,2022,38(02):213-216.doi:10.13329/j.cnki.zyyjk.2022.0058. In Chinese.

[22] Cheng RD, Dai Y, Li Y, et al.Etiological characteristics of infectious diarrhea in children[J]. J Pathogen Biol,2022,17(12):1425-1428,1434.doi:10.13350/j.cjpb.221212. In Chinese.

[23] Duan JQ, Wu XP, Yang YT,et al.Analysis of etiological surveillance results of 350 children with diarrhea[J]. J Pathogen Biol,2022,17(04):435-438.doi:10.13350/j.cjpb.220412. In Chinese.

[24] Han SY, Zhong L, Du SJ, et al. Analysis of surveillance results of viral diarrhea from one sentinel hospital in Haidian district of Beijing, 2012－2019[J].Capital J Public Health,2022,16(03):169-172. doi:10.16760/j.cnki.sdggws.2022.03.006. In Chinese.

[25] He F, Yin WL, Yang PF, et al. Analysis of the pathogen spectrum of viral diarrhea and the molecular epidemiological characteristics of norovirus in Huai'an[J]. J Pathogen Biol, 2022,17(06):631-634,642. doi:10.13350/j.cjpb.220603. In Chinese.

[26] Luo LF, Wang XG, Yan HJ,et al. Pathogenic surveillance of viral diarrhea in Minhang District of Shanghai in 2014 － 2020[J].J Pub Health and Prev Med,2022,33(03):76-80. In Chinese.

[27] Ma XZ, Cao RR, Liao XC,et al.Pathogen spectrum analysis of viral diarrhea in children under 5 years old in a sentinel hospital in Chengdu from 2011 to 2020[J].International J Virol, 2022,29(2):113-118.doi:10.3760/cma.j.issn.1673-4092.2022.02.006. In Chinese.

[28] Mao JW, Yang YL, Shi CC,et al. Molecular epidemiological characteristics of the virus in 96 children with acute diarrhea in Changdu of Tibet, China[J].Chin J Contemp Pediatr, 2022,24(03):266-272. In Chinese.

[29] Shabiremu Tohetamu, Zikemu Dilimurati, Nie JY,et al.Analysis of four kinds of virus infections and aetiology in infants and youngchildren in Urumqi from 2018 to 2020[J].Chin J Microecol,2022,34(04):420-425. doi:10.13381/j.cnki.cjm.202204008. In Chinese.

[30] Yao SP, Huang ZX, Zeng FY, et al. Etiology and epidemiological characteristics of infectious diarrhea，Qianjiang, Chongqing[J].Chin Frontier Health Quarantine,2022,45(02):167-169. doi:10.16408/j.1004-9770.2022.02.021. In Chinese.

[31] Tang X, Hu Y, Zhong X, et al. Molecular Epidemiology of Human Adenovirus, Astrovirus, and Sapovirus Among Outpatient Children With Acute Diarrhea in Chongqing, China, 2017-2019. Front Pediatr. 2022;10:826600. doi: 10.3389/fped.2022.826600.

[32] Wang G, Zhao RQ, Tang X, et al. Age-specific spectrum of etiological pathogens for viral diarrhea among children in twelve consecutive winter-spring seasons (2009-2021) in China. J Med Virol. 2022;94(8):3840-3846. doi: 10.1002/jmv.27790.

[33] Sun Z, Xu J, Niu P, et al. Aetiological Characteristics of Infectious Diarrhea in Yantai City, Shandong Province, China in 2017. Viruses. 2022;14(2):216. doi: 10.3390/v14020216.

[34] Liu LJ, Liu W, Liu YX, et al. Identification of norovirus as the top enteric viruses detected in adult cases with acute gastroenteritis. Am J Trop Med Hyg. 2022;82(4):717-22. doi: 10.4269/ajtmh.2010.09-0491.

[35] Cao YH, Yang JH, Jiang LL, et al.Analysis of pathogen detection of viral diarrhea in Southwest China from 2017 to 2019[J]. J Pub Health and Prev Med,2021,32(1):10-13. doi:10.3969/j.issn.1006-2483.2021.01.003. In Chinese.

[36] Hu JY, Yang JH, Cao YH, et al. Etiologies monitoring of viral diarrhea of children in Kunming[J].China J Trop Med,2021,21(03):234-237.doi:10.13604/j.cnki.46-1064/r.2021.03.07. In Chinese.

[37] Hu Y. Epidemiology of adenovirus, astrovirus and Sapovirus in children with acute diarrhea in Chongqing, 2017 to 2019[D]. Chongqing Medical University, 2022. Dissertation. doi:10.27674/d.cnki.gcyku.2021.000607. In Chinese.

[38] Jin D. Study on epidemiological characteristics of foodborne disease surveillance and outbreak events in Nanjing[D]. Southeast University, 2022. Dissertation. doi:10.27014/d.cnki.gdnau.2021.000887. In Chinese.

[39] Kuang XZ, Xiao WJ, Liu JC,et al. Etiological characteristics of viral gastroenteritis in pediatric inpatients under five years old in Shanghai, 2018–2020[J].Dis surveill, 2021,36(10):1069-1074. In Chinese.

[40] Li J, Xing Y, Zou WJ, et al. Etiological and epidemiological analysis of viral diarrhea surveillance in Hubei Province in 2017－2019[J].J Pub Health and Prev Med,2021,32(02):30-33. In Chinese.

[41] Yuan Y. Study on the Epidemiology Characteristics of Diarrhea Syndrome and Prediction of the Main Pathogen Infection among under-five Children in Gansu Province[D].Lanzhou University,2022.Dissertation.doi:10.27204/d.cnki.glzhu.2021.003668. In Chinese.

[42] Zhang WW, Feng BL, Wang JL, et al. Epidemiological characteristics of diarrhea pathogens and drug resistance of diarrhea-related bacteria in diarrheal patients in Miyun district, Beijing[J].Dis surveill,2021,36(04):381-386. In Chinese.

[43] Cao RR, Ma XZ, Li WY, et al. Epidemiology of norovirus gastroenteritis in hospitalized children under five years old in western China, 2015-2019. J Microbiol Immunol Infect. 2021;54(5):918-925. doi: 10.1016/j.jmii.2021.01.002.

[44] Luo X, Deng JK, Mu XP, et al. Detection and characterization of human astrovirus and sapovirus in outpatients with acute gastroenteritis in Guangzhou, China. BMC Gastroenterol. 2021;21(1):455. doi: 10.1186/s12876-021-02044-5.

[45] Chang H, Guo J, Wei Z, et al. Aetiology of acute diarrhoea in children in Shanghai, 2015-2018. PLoS One. 2021;16(4):e0249888. doi: 10.1371/journal.pone.0249888.

[46] Bi WJ, Xu JJ. Distribution of viral pathogens among child outpatients with acute diarrhea,2015-2018[J].Chin J Public Health,2020,36(9):1371-1373.doi:10.11847/zgggws1124618. In Chinese.

[47] Li XY, Hu X, Yue MN,et al. Pathogenic characteristics of viral diarrhea in children in Hangzhou during 2017-2018[J]. International Journal of Epidemiology and Infectious Diseases, 2020,47(2):128-132. doi:10.3760/cma.j.cn331340-20190731-00138. In Chinese.

[48] Shang XC, Shuai HQ, Zhao XQ,et al.Etiological and epidemiological characteristics of infant viral diarrhea in Hangzhou from 2012 to 2019[J].Chin J Dis Control Prev,2020,24(09):1110-1112. doi:10.16462/j.cnki.zhjbkz.2020.09.024. In Chinese.

[49] Su C. Pathogen spectrum and related factors of acute infectious diarrhea in Tianjin during 2017-2019 [D]. Tianjin Medical University, 2021. Dissertation. doi:10.27366/d.cnki.gtyku.2020.000505. In Chinese.

[50] Zhang WQ, Zhang HN, Han Y, et al. Etiology and epidemiological characteristics of gastroenteritis virus in food-borne diarrhea from three cities in Shandong Province, 2017[J].China J Prev Med,2020,54(2):169-174. doi:10.3760/cma.j.issn.0253-9624.2020.02.011. In Chinese.

[51] Wu L, Teng Z, Lin Q, et al. Epidemiology and Genetic Characterization of Classical Human Astrovirus Infection in Shanghai, 2015-2016. Front Microbiol. 2020;11:570541. doi: 10.3389/fmicb.2020.570541.

[52] Wang JX, Zhou HL, Mo ZJ, et al. Burden of viral gastroenteritis in children living in rural China: Population-based surveillance. Int J Infect Dis. 2020;90:151-160. doi: 10.1016/j.ijid.2019.10.029.

[53] Zeng H, Wang XN, Guan XH, et al. Analysis of the pathogenic and epidemiological characteristics of viral diarrhea in Hubei Province from 2016 to 2017[J].Mod Prev Med,2019,46(10):1737-1740. In Chinese.

[54] Gao L, Li JM. Pathogenic spectrum and epidemiological characteristics of diarrhea patients in Tianjin during 2015-2017[J]. Chin J Infect Dis,2019,37(4):209-213. doi:10.3760/cma.j.issn.1000-6680.2019.04.004. In Chinese.

[55] Guan HS, Sha D, Feng WH, et al. Etiological surveillance results of infectious diarrhea in Wuxi, Jiangsu, 2014 –2018[J].Dis surveill,2019,34(09):795-799. In Chinese.

[56] Lin L, Fu ZY, Li JS, et al. Analysis of surveillance results of viral diarrhea among children under 5 years of age in Shandong province from 2012 to 2017[J].Chinese J Exp Clin Virol,2019,33(5):463-467.doi:10.3760/cma.j.issn.1003-9279.2019.05.004. In Chinese.

[57] Wang F, Jin M, Li DD, et al.Molecular epidemiology of viral diarrhea in children at ages of less than 5 years in Lanzhou City, Gansu Province, China in 2017[J].Chin J Biologicals, 2019,32(10):1102-1107. doi:10.13200/j.cnki.cjb.002833. In Chinese.

[58] Yuan JM, Zhang F, Wei Y, et al. Analysis of virological results of children with infectious diarrhea under 5 years old in Nantong city[J]. Journal of Xinjiang Medical University, 2019,42(06):828-832. In Chinese.

[59] Zhang HL, Ye YH, He YQ, et al.The characteristics of sapovirus infection among patients with diarrhea in Shenzhen in 2016[J].International J Virol,2019,26(1):31-34. doi:10.3760/cma.j.issn.1673-4092.2019.01.09. In Chinese.

[60] Zhang JY, Liu XX, Chu YH, et al. Surveillance of viral diarrhea in children under 5 years old in Xicheng District, Beijing, 2018[J].J Pub Health and Prev Med,2019,30(06):59-62. In Chinese.

[61] Xue L, Cai W, Zhang L, et al. Prevalence and genetic diversity of human sapovirus associated with sporadic acute gastroenteritis in South China from 2013 to 2017. J Med Virol. 2019;91(10):1759-1764. doi: 10.1002/jmv.25511.

[62] Chen C, Wang LP, Yu JX, et al. Prevalence of Enteropathogens in Outpatients with Acute Diarrhea from Urban and Rural Areas, Southeast China, 2010-2014. Am J Trop Med Hyg. 2019;101(2):310-318. doi: 10.4269/ajtmh.19-0171.

[63] Cheng SS, Dong Y, Liu GL, et al. Etiological analysis of viral diarrhea among preschoolers in Shijiazhuang from 2012 to 2017[J]. J Pathogen Biol,2018,13(12):1389-1392,1396. doi:10.13350/j.cjpb.181221. In Chinese.

[64] Gong XH, Wu HY, Xiao WJ, et al. Surveillance of infectious diarrhea patients in Shanghai during 2013-2016 ,based on establishment of diarrhea public health comprehensive surveillance system[J].Chin J Infect Dis,2018,36(6):327-332.doi:10.3760/cma.j.issn.1000-6680.2018.06.002. In Chinese.

[65] Gong CH, Liu F, Chen M, et al. Surveillance and analysis of pathogen spectrum of diarrhea in children in Sanlin area of Shanghai from 2015 to 2016[J].Pract Prev Med,2018,25(7):886-889.doi:10.3969/j.issn.1006-3110.2018.07.035. In Chinese.

[66] Guo LM, Guo S, Liu L, et al. Monitoring and analysis of pathogenic microorganisms in 463 patients with diarrhea[J]. J Pathogen Biol,2018,13(05):536-538,542.doi:10.13350/j.cjpb.180519. In Chinese.

[67] Jiang X, Du XF, Ye YH, et al. Analysis of pathogen surveillance results for diarrhoea in children from 2015 to 2016 in Nanjing[J].Pract Prev Med,2018,25(12):1508-1510. In Chinese.

[68] Jin J, Zhang YC, Liu YL, et al. Etiology of virus caused diarrhea in children aged ＜5 years in Wuhan, 2015 [J].Dis surveill,2018,33(11):940-944. In Chinese.

[69] Luo KW, Zhao SL, Hu SX, et al. Pathogen spectrum distribution of infectious diarrhea in Hunan province from 2015 to 2017 [J].Journal of Tropical Diseases and Parasitology, 2018,16(02):71-74. In Chinese.

[70] Tan WW, Zhang WB, Xu HY, et al. Analysis of surveillance results of viral diarrhea of Nantong City in 2017[J].Mod Prev Med,2018,45(20):3803-3806. In Chinese.

[71] Zhang YL, Li J, Li M, et al. Composition of pathogens on viral diarrhea in Yichang（2017）[J]. J Pub Health and Prev Med,2018,29(06):90-93. In Chinese.

[72] He LY, Zhou YM, Gu WP, et al. Molecular epidemiological characteristics of human calicivirus in Kunming from 2014 to 2015 [J].Chinese J Exp Clin Virol,2017,31(6):525-529. doi:10.3760/cma.j.issn.1003-9279.2017.06.009. In Chinese.

[73] Su T, Liu YY, Yu QL, et al. Infection status and epidemiology of Human calicivirus（HuCV) among children under 5 years of age in Sentinel hospitals in Hebei Province,2010-2015[J]. J Pathogen Biol,2017,12(12):1188-1191,1214.doi:10.13350/j.cjpb.171213. In Chinese.

[74] Sun JS, Yuan JC, Wang XK, et al. Analysis of etiological surveillance results of diarrhea disease in Jinshan District of Shanghai in recent 2 years[J].Shanghai J Prev Med,2017,29(03):209-211.doi:10.19428/j.cnki.sjpm.2017.03.013. In Chinese.

[75] Wang HY. Molecular epidemiology of noroviruses based on the sentinel hospitals surveillance of foodborne disease in Shandong province[D].Shandong University,2018.

Dissertation. In Chinese.

[76] Xie SR, Shen HW, Zhang JJ, et al. Etiology characteristics and epidemiological analysis of viral diarrhea in Shenzhen, 2014-2015[J].China J Trop Med,2017,17(07):686-690. doi:10.13604/j.cnki.46-1064/r.2017.07.11. In Chinese.

[77] Yuan L, Pan XP, Liu GX, et al. Analysis of viral diarrhea in children under 5 years old in Xining from 2015 to 2016[J].J Med Pest Control,2018,34(02):147-150. In Chinese.

[78] Zhou Y, Qi L, Liang JR, et al. Etiological study of viral diarrhea among children under 5 years of age in Dongcheng district of Beijing[J]. International J Virol, 2017,24(2):119-122. doi:10.3760/cma.j.issn.1673-4092.2017.02.012. In Chinese.

[79] Chen WB, Yu F, Qiao YQ, et al. The prevalence of astrovirus, sapovirus, and adenovirus enteric infections in children with acute diarrhea in Chongqing in 2014[J]. J Clin Pediatr ,2016,34(04):241-245. In Chinese.

[80] Ding M, Liu CL, Pan LF, et al. Detection and analysis of viral diarrhea in Shanghai Pudong New District from 2012 to 2013[J].J Navy Med,2016,37(03):233-236. In Chinese.

[81] Pang BB, Zhou X, Nobumichi Kobayashi, et al. Surveillance and analysis of the pathogens of viral gastroenteritis in 2011 and 2013, Wuhan,China[J].Chin J Zoonoses,2016,32(05):457-461. In Chinese.

[82] Su J, Xu HY, Xiong HP. Results of virological surveillance on infectious diarrhea in Nantong City from 2012-2014[J]. Occup and Health, 2016,32(22):3080-3082,3087. doi:10.13329/j.cnki.zyyjk.2016.0972. In Chinese.

[83] Wang DY, Wang J, Chang WB, et al. Active surveillance of foodborne diseases in Changshu City from 2012 to 2015[J].J Pub Health and Prev Med,2016,27(02):71-73. In Chinese.

[84] Xu ZQ, Chen JJ, Zhu SQ, et al. The epidemiology of adults acute viral gastroenteritis in Shanghai Changning district from 2010 to 2013[J].Chinese Journal of Advanced Medical Education, 2016,39(5):419-424.doi:10.3760/cma.j.issn.1673-4904.2016.05.009. In Chinese.

[85] Zhao JY, Shen XJ, Zhang BF, et al. Etiological agents distribution and epidemiology of viral diarrhea in children below 5 years old in He′nan province, 2008-2015[J].Chin J Infect Dis,2016,34(12):738-742. doi:10.3760/cma.j.issn.1000-6680.2016.12.008. In Chinese.

[86] Zheng SF, Yu F, Chen X, et al. Surveillance on pathogens of acute diarrhea in sentinel hospitals in Zhejiang province from 2009 to 2014 [J].China J Prev Med,2016,50(12):1084-1090. doi:10.3760/cma.j.issn.0253-9624.2016.12.012. In Chinese.

[87] Zhou YK, Jin M, Kong XY, et al. The analysis of sapovirus infection in children under 5 years old with diarrhea in eight provinces of China, 2012-2014[J].International J Virol,2016,23(2):81-84.doi:10.3760/cma.j.issn.1673-4092.2016.02.003. In Chinese.

[88] Shen H, Zhang J, Li Y, et al. The 12 Gastrointestinal Pathogens Spectrum of Acute Infectious Diarrhea in a Sentinel Hospital, Shenzhen, China. Front Microbiol. 2016;7:1926. doi: 10.3389/fmicb.2016.01926.

[89] Zhang SX, Li L, Yin JW, et al. Emergence of human caliciviruses among diarrhea cases in southwest China. BMC Infect Dis. 2016;16(1):511. doi: 10.1186/s12879-016-1831-5.

[90] Li LL, Liu N, Humphries EM, et al. Aetiology of diarrhoeal disease and evaluation of viral-bacterial coinfection in children under 5 years old in China: a matched case-control study. Clin Microbiol Infect. 2016;22(4):381.e9-381.e16. doi: 10.1016/j.cmi.2015.12.018.

[91] Dong HY, Ma ZL, Yang JG. Aetiological study on viral pathogens of diarrhea patients from Taizhou in 2013[J].Mod Prev Med,2015,42(01):134-136. In Chinese.

[92] Hong ZT, Xu L, Zhong HL,et al.Etiological study on diarrhea virus in Guangzhou area from 2011 to 2013[J].J Trop Med,2015,15(12):1679-1682. In Chinese.

[93] Jin M, Li HY, Kong XY, et al. Detection and typing assay of sapovirus in adults with acute gastroenteritis from 2008 to 2009 in Beijing[J].Chin J Exp Clin Virol,2015,29(1):62-64.doi:10.3760/cma.j.issn.1003-9279.2015.01.021. In Chinese.

[94] Luo X. Development and application of a multiplex RT-PCR method detected 5 types of diarrhea-related virus and Investigation of diarrhea pathogens in Guangzhou from September 2013 to October 2014[D].Southern Medical University,2016.Dissertation. In Chinese.

[95] Xie J. Epidemiologic study for viral infantile diarrhea in Lanzhou area in 2013-2014[D]. Lanzhou University,2016.Dissertation. In Chinese.

[96]Yu JX. Pathogenic spectrum characteristics and estimated incidence of diarrhea[D].China CDC,2022.Dissertation.doi:10.27511/d.cnki.gzyyy.2015.000002. In Chinese.

[97] Zhang M, Sun CG, Chen X, et al. Pathogen spectrum analysis of infectious diarrhea in Hangzhou during 2013[J]. Chin J Clin Lab Sci, 2015,33(06):469-471. doi:10.13602/j.cnki.jcls.2015.06.21. In Chinese.

[98] Zhang YH, Xue N, Liu ZL, et al. Analysis of pathogen detection and epidemic characteristics of 208 children with viral diarrhea disease[J].Journal of Ningxia Medical University,2015,37(12):1436-1438.doi:10.16050/j.cnki.issn1674-6309.2015.12.018. In Chinese.

[99] Zhang DM, Ma MM, Wen WT, et al. Clinical epidemiology and molecular profiling of human bocavirus in faecal samples from children with diarrhoea in Guangzhou, China. Epidemiol Infect. 2015;143(11):2315-29. doi: 10.1017/S0950268814003203.

[100] Lu L, Jia R, Zhong H, et al. Molecular characterization and multiple infections of rotavirus, norovirus, sapovirus, astrovirus and adenovirus in outpatients with sporadic gastroenteritis in Shanghai, China, 2010-2011. Arch Virol. 2015;160(5):1229-38. doi: 10.1007/s00705-015-2387-1.

[101] Li K, Zhang C, Zhao R, et al. The prevalence of STL polyomavirus in stool samples from Chinese children. J Clin Virol. 2015;66:19-23. doi: 10.1016/j.jcv.2015.02.017.

[102] Chen HF, Hu TT, Yao YX, et al. Etiological and epidemic characterization of viral diarrhea in children under the age of 5 years in Guangzhou City[J]. Chin J Dis Control Prev, 2014,18(4):336-339. In Chinese.

[103] Hong WS, Liao XW, Liu MZ, et al. Etiological surveillance of infectious diarrhea in outpatients of Ruian People's Hospital[J].Shanghai J Prev Med,2014,26(07):362-364. doi:10.19428/j.cnki.sjpm.2014.07.005. In Chinese.

[104] Li RQ, Hua WY ,Sun YM, et al. Etiology investigation and epidemiological analysis of viral diarrhea among adults in Haidian district, beijing[J].Mod Prev Med,2014,41(23):4235-4237,4251. In Chinese.

[105] Qiao K, Luo LF, Song CP, et al. Etiological study on enteric viruses in adult diarrhea in the southwest of Shanghai[J]. Occup and Health,2014,30(14):2012-2014. doi:10.13329/j.cnki.zyyjk.2014.14.047. In Chinese.

[106] Xiang JY. Epidemiological study on viral diarrhea among children in Lanzhou[D].Lanzhou University,2015.Dissertation. In Chinese.

[107] Zhao J. Epidemiological and genetic features of gastrointestinal viruses in pediatric outpatients with acute diarrhea in Chongqing[D].Academy of Military Medical Sciences,2015.

Dissertation. In Chinese.

[108] Wu W, Yang H, Zhang HL, et al. Surveillance of pathogens causing gastroenteritis and characterization of norovirus and sapovirus strains in Shenzhen, China, during 2010. Arch Virol. 2014;159(8):1995-2002. doi: 10.1007/s00705-014-1986-6.

[109] Wang G, Shen Z, Qian F, et al. Genetic diversity of sapovirus in non-hospitalized adults with sporadic cases of acute gastroenteritis in Shanghai, China. J Clin Virol. 2014;59(4):250-4. doi: 10.1016/j.jcv.2014.01.007.

[110] An SY, Zhao Z, Guo JQ, et al. Epidemiological study on viral diarrhea during 2009-2011 in Liaoning Province[J]. Chin J Infect Dis,2013,31(3):166-169. doi:10.3760/cma.j.issn.1000-6680.2013.03.007. In Chinese.

[111] Fei Y, Sun Q, Fu YF, et al. Surveillance and analysis on diarrheal disease pathogen spectrum among children under 5 years old in Pudong New Area of Shanghai City[J].Shanghai J Prev Med,2013,25(11):602-605.doi:10.19428/j.cnki.sjpm.2013.11.002. In Chinese.

[112] Hu TT. The epidemiological study of viral diarrhea in sentinelhospitais of Guangzhou from 2011-2012[D].Southern Medical University,2014.Dissertation. In Chinese.

[113] Lu LJ. Molecular Epidemiology of Viruses causing Acute Diarrheain Children in Shanghai between 2006 and 2011[D].Fudan University,2013.Dissertation. In Chinese.

[114] Miu GZ, Ma Y, Lu HD, et al. Investigation on infectious diarrhea pathogens and epidemiological characteristics in Jiangyin city [J].J Pub Health and Prev Med,2013,24(05):37-40. In Chinese.

[115] Zikemu Dilimurati, Ayguri Ilhali, Liu HB,et al. Surveillance of viral diarrhea among hospitalized children in Urumqi, 2010[J].Dis surveill,2013,28(06):439-442. In Chinese.

[116] Ye HY, Zhou FM, Cui DW, et al. Distribution and clinical features of gastrointestinal virus infection in infants with acute diarrhea [J].Chinese Journal of Clinical Infectious Diseases, 2013,6(6):335-338. doi:10.3760/cma.j.issn.1674-2397.2013.06.004. In Chinese.

[117] Chen SY, Tsai CN, Chen CL, et al. Severe viral gastroenteritis in children after suboptimal rotavirus immunization in Taiwan. Pediatr Infect Dis J. 2013;32(12):1335-9. doi: 10.1097/INF.0b013e3182a5f5b6.

[118] Chen Y, Li Z, Han D, et al. Viral agents associated with acute diarrhea among outpatient children in southeastern China. Pediatr Infect Dis J. 2013;32(7):e285-90. doi: 10.1097/INF.0b013e31828c3de4.

[119] Li XL, Li DD, Cheng WX, et al. Molecular and epidemiological study on among children under 5 years old in Nanjing[J].Chinese J Exp Clin Virol,2012,26(1):14-17. doi:10.3760/cma.j.issn.1003-9279.2012.01.006. In Chinese.

[120] Li XY. Etiological Study on Viral Diarrhea among Patients under Five Years Old in Sentinel Hospital in Hebei 2010[D].Hebei Medical University,2012.Dissertation. In Chinese.

[121] Lin Q, Jin Y, Zhou JS, et al. Molecular epidemiological study on viral diarrhea among pediatric patients under five years old in Nanjing, 2009－2010[J].Chin J Evid Based Pediatr,

2012,7(01):31-36. In Chinese.

[122] Wang YX. Epidemiological Study on Common DiarrhealViruses among Infants and Young Childrenin Lanzhou from 2010 to 2011[D].Lanzhou University,2012.Dissertation. In Chinese.

[123] Liu ZH, Gong ST. Prevalence of sapoviral infection in a children’s hospital of Guangzhou[J]. J Clin Pediatr ,2011,29(06):514-517. In Chinese.

[124] Tan DM, Liu W, Deng LL,et al. Sapovirus infection among sporadic adult cases with acute gastroenteritis in Nanning City[J].Chin J Zoonoses,2011,27(04):294-296,306. In Chinese.

[125] Wang YG. Molecular epidemiology characteristics of caliciviruses in patients with diarrhea in Shenzhen, 2009[D].Southern Medical University,2012.Dissertation. In Chinese.

[126] Zhu-ge XL. Molecular Epidemiological Study on HumanCaliciviruses of Acute Diarrhea Patients in Zhejiang Province[D].Zhejiang University,2011.Dissertation. In Chinese.

[127] Zhu-ge XL, Cui DW, Wu YP, et al. Detection and typing of caliciviruses from patients with acute diarrhea in Hangzhou area,2009-2010[J].Chin J Epidemiol,2011,32(10):1022-1025. doi:10.3760/cma.j.issn.0254-6450.2011.10.016. In Chinese.

[128] Gong ZX.Molecular epidemiology of Viral diarrhea among children in Shanghai[D].The Second Military Medical University,2010.Dissertation. In Chinese.

[129] Li BW. Molecular and epidemiological study on viral diarrhea among infants and young children in Lanzhou[D].Lanzhou University,2010.Dissertation. In Chinese.

[130] Li YQ. Molecular epidemiological study on human caliciviruses ofhospital children with diarrhea under 5 years of agein Gansu province of China[D].China CDC,2011.Dissertation. In Chinese.

[131] Chang ZR, Jin M, Liu N, et al. Epidemiological and genetic analysis of Sapovirus in nine provinces of China in 2006[J]. Chin J of Virol,2009,25(2):113-116. doi:10.3321/j.issn:1000-8721.2009.02.006. In Chinese.

[132] Jin Y, Cheng WX, Yang XM, et al. Viral agents associated with acute gastroenteritis in children hospitalized with diarrhea in Lanzhou, China. J Clin Virol. 2009;44(3):238-41. doi: 10.1016/j.jcv.2008.12.010.

[133] Cheng WX. Epidemiological Study on Viral Diarrhea among Infantsand Young Children in Lanzhou[D]. Lanzhou University, 2009. Dissertation. In Chinese.

[134] Ye XH, Jin Y, Fang ZY, et al. Infection of sapoviruses children hospitalized with acute gastroenteritis in Lanzhou 2003 - 2007[J].Chin J Epidemiol,2008,29(8):843-844. In Chinese.

[135] Sun YP, Hiroshi Ushijima, Xie HP, et al. Application of typing primer RT-PCR in molecular epidemiology of calicivirus[J].International J Virol,2006(05):129-133. In Chinese.

**Outbreak studies:**

[1] Chen XB,Chen XX,Li DF,et al. Investigation of an outbreak of sapovirus infection in a middle school in Chaozhou City, Guangdong Province in 2022 [J]. China Tropical Medicine, 2024,24(05):603-607. In Chinese.

[2] Tian J, Cong S, Chen L,et al. Molecular etiological study on the GI.6 Norovirus causing an infectious diarrhea outbreak in Shenyang, China [J]. Chinese J Exp Clin Virol,2023,37(3):310-314. doi:10.3760/cma.j.cn112866-20230403-00033. In Chinese.

[3] Shi RJ, Yang M, Xu YC,et al. Investigation of an epidemic of acute gastroenteritis caused by Sapovirus in a middle school [J]. J Med Pest Control, 2023,39(10):998-1002,1007. In Chinese.

[4] Yao P, Li Q, Jiang X,et al. Epidemiological and genetic characteristics of sapovirus clusters in Changzhou schools from 2019 to 2022 [J]. Chin J Sch Health, 2023, 44(10):1574-1577. doi:10.16835/j.cnki.1000-9817.2023.10.030. In Chinese.

[5] Wang P, Wang XY, Kang Q,et al. Infant infection with sarovirus GⅠ.2 in Gansu: an investigation and analysis of clustered epidemic situation [J]. Dis surveill, 2023,38(02):233-236. In Chinese.

[6] Su L, Mao H, Sun Y, et al. The analysis of the genotype of Sapovirus outbreaks in Zhejiang Province. Virol J. 2023;20(1):268. doi: 10.1186/s12985-023-02202-z.

[7] Zou L, Li Y, Zhou G, et al. A Large Acute Gastroenteritis Outbreak Associated with Both Campylobacter coli and Human Sapovirus - Beijing Municipality, China, 2021. China CDC Wkly. 2023;5(52):1167-1173. doi: 10.46234/ccdcw2023.219.

[8] Feng TC, Hu YD, Mao JY,et al. Epidemiological characteristics of Sapovirus virus clustered vomiting epidemic in Baoshan District in Shanghai from 2017 to 2019 [J]. Chin J Sch Health,2022,43(12):1809-1811,1816.

[9] Li DL, Zeng Z, Zhang WY,et al. Investigation of an epidemic of sapovirus infection in an university [J]. Dis surveill, 2022,37(04):553-556. In Chinese.

[10] Zhu L, Dong ZP, Du JP,et al.Epidemiological characterization of sapovirus gastroenteritis aggregation in a kindergarten in Jinshan District, Shanghai [J]. Shanghai J Prev Med, 2021,33(06):521-524. doi:10.19428/j.cnki.sjpm.2021.19643. In Chinese.

[11] Wu FT, Oka T, Kuo TY, et al. Sapoviruses detected from acute gastroenteritis outbreaks and hospitalized children in Taiwan. J Formos Med Assoc. 2021;120(8):1591-1601. doi: 10.1016/j.jfma.2020.11.020.

[12] Li JY, Mai W, Tan HQ,et al. An outbreak of acute gastroenteritis caused by Sapovirus in a community of Guangdong province [J]. Chin J Epidemiol, 2020,41(2):226-230. doi:10.3760/cma.j.issn.0254-6450.2020.02.016. In Chinese.

[13] Li Y. Investigated a cluster of acute gastroenteritis with mixed infections together with kindergarten astroviruses and zoonotic viruses[J].South China J Prev Med,2020,46(06):650-652. In Chinese.

[14] Guo L, Shao YP, Chen CZ, et al. Epidemiological characteristics of the cluster of Zaru virus in Haidian District, Beijing from 2015 to 2018 [J]. Pract Prev Med, 2020,27(01):91-93. In Chinese.

[15] Yan Y, Li Y, Shi W, et al. An outbreak of gastroenteritis associated with a novel GII.8 sapovirus variant-transmitted by vomit in Shenzhen, China, 2019. BMC Infect Dis. 2020;20(1):911. doi: 10.1186/s12879-020-05643-x.

[16] Huang SY, He JF, Yi LP, et al.Outbreak investigation of acute gastroenteritis by secondary pollution in water supply，Pingxiang College，Jiangxi [J]. Mod Prev Med, 2020,47(03):542-545. In Chinese.

[17] Huang YF, Yuan M, Xu YZ, et al. Epidemiology of an acute gastroenteritis outbreak caused by sapovirus in a primary school in Shenzhen, Guangdong,2018 [J]. Dis surveill, 2019,34(07):676-679. In Chinese.

[18] Xing Y, Hua WY, Chen CZ,et al. Investigation of an acute gastroenteritis outbreak caused by sapovirus in a kindergarten [J]. International J Virol, 2018,25(3):185-188. doi:10.3760/cma.j.issn.1673-4092.2018.03.009. In Chinese.

[19] Wang Md J, Li PhD Y, Kong Md X, et al. Two gastroenteritis outbreaks caused by sapovirus in Shenzhen, China. J Med Virol. 2018;90(11):1695-1702. doi: 10.1002/jmv.25236.

[20] Long DL, Zhuang HY, Jin M,et al. Molecular epidemiology of an acute gastroenteritis outbreak caused by sapovirus [J]. International J Virol, 2017,24(3):183-186. doi:10.3760/cma.j.issn.1673-4092.2017.03.009. In Chinese.

[21] Wang Y, Zhang J, Shen Z. The impact of calicivirus mixed infection in an oyster-associated outbreak during a food festival. J Clin Virol. 2015;73:55-63. doi: 10.1016/j.jcv.2015.10.004.

[22] Xu CH, Wang Z, Wang J. Investigation on a cluster of acute gastroenteritis caused by Javirus in primary school [J]. Chin J Disinfection, 2014,31(05):490-492. In Chinese.

[23] He YQ, Zhuo F, Zhang HL,et al. Molecular epidemiology of a cute gastroenteritis outbreak caused by Sapovirus in Adults [J]. Dis surveill,2012,27(02):101-103. In Chinese.

[24] Wu FT, Oka T, Takeda N, et al. Acute gastroenteritis caused by GI/2 sapovirus, Taiwan, 2007. Emerg Infect Dis. 2008;14(7):1169-71. doi: 10.3201/eid1407.071531.
